# Elevated miR-130a/miR130b/miR-152 expression reduces intracellular ATP levels in the pancreatic beta cell

**DOI:** 10.1038/srep44986

**Published:** 2017-03-23

**Authors:** Jones K. Ofori, Vishal A. Salunkhe, Annika Bagge, Neelanjan Vishnu, Mototsugu Nagao, Hindrik Mulder, Claes B. Wollheim, Lena Eliasson, Jonathan L. S. Esguerra

**Affiliations:** 1 Islet Cell Exocytosis, Department of Clinical Sciences-Malmö, Lund University, Malmö, 205 02, Sweden; 2 Lund University Diabetes Centre, Skåne University Hospital, Lund and Malmö, Sweden; 3 Molecular Metabolism, Department of Clinical Sciences-Malmö, Lund University, Malmö, 20502, Sweden; 4 Department of Cell Physiology and Metabolism, Faculty of Medicine, University of Geneva, Geneva, 1211, Switzerland

## Abstract

MicroRNAs have emerged as important players of gene regulation with significant impact in diverse disease processes. In type-2 diabetes, in which impaired insulin secretion is a major factor in disease progression, dysregulated microRNA expression in the insulin-secreting pancreatic beta cell has been widely-implicated. Here, we show that miR-130a-3p, miR-130b-3p, and miR-152-3p levels are elevated in the pancreatic islets of hyperglycaemic donors, corroborating previous findings about their upregulation in the islets of type-2 diabetes model Goto-Kakizaki rats. We demonstrated negative regulatory effects of the three microRNAs on pyruvate dehydrogenase E1 alpha (PDHA1) and on glucokinase (GCK) proteins, which are both involved in ATP production. Consequently, we found both proteins to be downregulated in the Goto-Kakizaki rat islets, while *GCK* mRNA expression showed reduced trend in the islets of type-2 diabetes donors. Overexpression of any of the three microRNAs in the insulin-secreting INS-1 832/13 cell line resulted in altered dynamics of intracellular ATP/ADP ratio ultimately perturbing fundamental ATP-requiring beta cell processes such as glucose-stimulated insulin secretion, insulin biosynthesis and processing. The data further strengthen the wide-ranging influence of microRNAs in pancreatic beta cell function, and hence their potential as therapeutic targets in type-2 diabetes.

Insulin secreted from the pancreatic beta cells is indispensable for maintaining glucose homeostasis in healthy individuals. The molecular events accounting for the insulin secretory response of beta cells to elevated blood glucose are called stimulus-secretion coupling. This process consists of key cellular events: glucose uptake and metabolism to elevate cytosolic ATP/ADP ratios, closure of K_ATP_ channels leading to membrane depolarization, and opening of voltage-sensitive calcium channels causing influx of calcium ions, which ultimately facilitates insulin granule exocytosis^1^. Other nutrients including amino acids and free fatty acids, as well as incretins such as glucagon-like peptide 1 (GLP-1), potentiate insulin secretion. All of these, with the exception of a few amino acids, require the presence of glucose, underlining the central role of mitochondrial glucose metabolism in insulin secretion^2^.

The deterioration of glucose-stimulated insulin secretion (GSIS) in the pancreatic beta cell is an early sign of type-2 diabetes (T2D), even preceding insulin resistance in the target tissues^3^. Indeed, genome-wide association studies (GWAS) implicate dozens of genes with important roles in pancreatic beta cell function^4^. Consequently, functional deficiencies in the processes of stimulus-secretion coupling ultimately cause defective insulin secretion. Although there is a canonical understanding of the biochemistry underlying stimulus-secretion coupling in the pancreatic beta cells, the various molecular genetic mechanisms regulating its individual components are incompletely understood.

The important roles and functional implications of non-coding RNAs in pancreatic beta cell development and physiology are widely recognized^5^,^6^. For instance, specific microRNAs (miRNAs) have been shown to be involved in the different aspects of GSIS^5^. Mature miRNAs generally bind the 3′UTR region, but may also bind within the coding sequence (CDS) of the target mRNA which leads to degradation, deadenylation and/or translational repression, with the net effect of reduced protein expression of the target^7^. The significance of miRNAs for maintaining beta cell identity is particularly highlighted by the contribution of miR-29a/b in the constitutive repression of the *MCT-1* (monocarboxylate transporter) gene. This gene transcribes the pyruvate/lactate transporter MCT-1, which is “disallowed/forbidden” in the beta cells to prevent muscle-derived pyruvate to stimulate insulin release during exercise^8^,^9^.

We previously showed dysregulated expression of many miRNAs in the pancreatic islets of Goto-Kakizaki (GK) rats^10^, a well-studied rodent model of T2D primarily characterized by impaired GSIS^11^. The polygenic effects from at least three *Niddm* (non-insulin dependent diabetes mellitus) loci were discovered to affect insulin release and cause hyperglycaemia^12^. Interestingly, the molecular lesions characterizing impaired GSIS in the GK beta cell were found to be diverse, ranging from decreased expression of certain components of the secretory machinery, *i.e.* exocytotic proteins^13^,^14^, perturbed adrenergic signalling^15^ and glucose metabolism^16^, to reduced activity of enzymes in specific biochemical pathways, *e.g.* deficient pyruvate dehydrogenase activity in mitochondria^17^.

The upregulated miRNAs in the GK islet can down-regulate the expression of exocytotic proteins, thereby leading to reduced insulin secretion and hyperglycaemia in the animals^10^. In addition, we found putative targets of upregulated GK islet miRNAs involved in other aspects of stimulus-secretion coupling. Here, we investigated the effect on GSIS of three upregulated GK islet miRNAs: miR-130a-3p (miR-130a), miR-130b-3p (miR-130b) and miR-152-3p (miR-152), in the context of cellular metabolism by direct measurement of cytosolic ATP in live single insulin beta cells using PercevalHR, a genetically-encoded fluorescent reporter of ATP:ADP ratio^18^,^19^,^20^.

We modulated the miRNA levels in the beta cell line, INS-1 832/13 and focused on gene targets relevant for ATP production: (i) the *Pdha1* gene, which codes for the E1 alpha subunit of the multi-enzyme complex pyruvate dehydrogenase (PDH) in the mitochondria, and (ii) the *Gck* (glucokinase) gene, which is the recognized “glucose-sensor” of pancreatic beta cells, a key regulating enzyme catalysing the phosphorylation of glucose as the first step of the glycolytic pathway^21^. We also investigated the effect of elevated levels of the miRNAs in known ATP-requiring processes such as in pro-insulin to insulin conversion^22^,^23^.

Specific deletion of *Pdha1* in mouse beta cells (β-PDHKO) results in deficiency in PDH activity, impaired GSIS and development of hyperglycaemia^24^. Regarding glucokinase, the heterozygote inactivating mutation in this gene is the first reported sub-type of the maturity-onset diabetes of the young (MODY) causing reduced insulin secretion, and hence hyperglycaemia^25^,^26^.

Here, corroborating our previous findings in the T2D model GK rat islets, we report the elevated expression of miR-130a, miR-130b and miR-152 in human islets from donors with impaired glucose tolerance (IGT) and T2D. We consequently found reduced expression in the protein level of both GCK and PDHA1 in the pancreatic islets of GK rats, while there was a trend of decreased *GCK* mRNA expression in islets from T2D donors. We then dissected how the miRNAs influence GSIS via their negative regulatory effects on glucose metabolism in the pancreatic beta cells. Specifically, we show that the negative regulation of *Pdha1* or *Gck* by the miRNAs, can partially account for the reduced cytosolic ATP observed in beta cells with abnormally high levels of miR-130a/b and/or miR-152. Overall, our results support the contribution of dysregulated miRNA expression to the impaired regulation of beta cell stimulus-secretion coupling, a hallmark of beta cell failure in the development of T2D.

## Results

### Upregulation of miR-152, miR-130a and miR-130b in GK rat islets, and in human islets from donors with impaired glucose tolerance and type-2 diabetes

**Figure 1:**
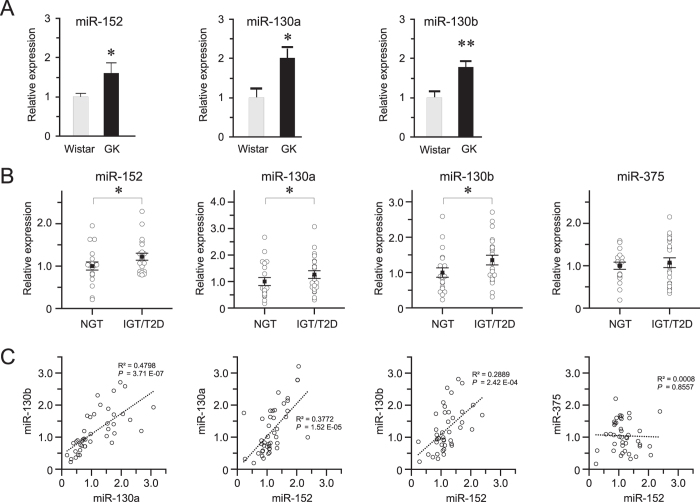
Over-expression and co-expression of miR-152, miR-130a, and miR-130b in pancreatic islets. **(A)** Validation of miR-152, miR-130a, and miR-130b upregulation in GK rat islets by qPCR. Expression of each miRNA was normalized to U87 rat and U6 snRNA expression. Wistar expression level was used as calibrator and relative quantification was done by using the ΔΔCt method. **(B)** Expression in the human pancreatic islets. (NGT = Normal glucose tolerance, IGT = Impaired glucose tolerance, T2D = Type-2 diabetes). RNU48 was used as the normalizer, and relative quantification by ΔΔCt method. **(C)** Co-expression analysis of miRNA expression using simple linear regression with F-test to determine significance at p < 0.05. Student’s t-test (unpaired, two-sided) was used to determine significance in Wistar (n = 4) vs GK (n = 11) rat islets. For the human qPCR data (NGT, n = 20; IGT/T2D, n = 22), Mann-Whitney U test (unpaired, one-sided) was used to determine the significance. Error bars where present are S.E.M. *p < 0.05, **p < 0.01.

We previously determined that miR-152 and miR-130a were among the 24 upregulated miRNAs in the islets of the T2D model GK rat, compared to those of Wistar controls using a locked nucleic acid (LNA)-based miRNA array profiling approach^10^. Here, we validated our array findings by qPCR, and showed that miR-130b, which harbours an identical seed sequence as miR-130a and belongs to the miR-130 gene family, was also upregulated in the pancreatic islets of hyperglycaemic GK rats (Fig. 1A).

We hypothesized that the pancreatic islets from hyperglycaemic human donors would likewise exhibit elevated levels of miR-152 and miR-130a/b and therefore grouped the human islets according to the glycated haemoglobin (% HbA1c) levels of the donors, *i.e.*, a long-term measure of glycaemia. The characteristics of human pancreatic islet donors are in Supplementary Table 1. We found that the levels of miR-152, miR-130a and miR-130b were upregulated in the islets of hyperglycaemic donors (IGT/T2D) compared to those of normoglycemic (NGT) donors (Fig. 1B). Moreover, despite different chromosomal locations of the three miRNAs in the human genome (chr11/miR-130a; chr22/miR-130b; chr17/miR-152), there was a notable co-expression among them, indicating highly-coordinated transcriptional regulation of these miRNAs in the human pancreatic islets (Fig. 1C).

### Effect of modulating miR-130a, miR-130b, and miR-152 levels on insulin secretion in INS-1 832/13 cells

To dissect the effect of the three miRNAs in pancreatic beta cell stimulus-secretion coupling, we performed transient over-expression or knockdown of the miRNAs using mature miRNA mimics or LNA anti-miRs, respectively, in INS-1 832/13 cells (Supplementary Fig. S1). We hereafter refer to over-expression and knockdown of specific miRNA with the prefix “OE” and “LNA”, respectively. The controls were scrambled oligonucleotides referred to as SCR.

**Figure 2:**
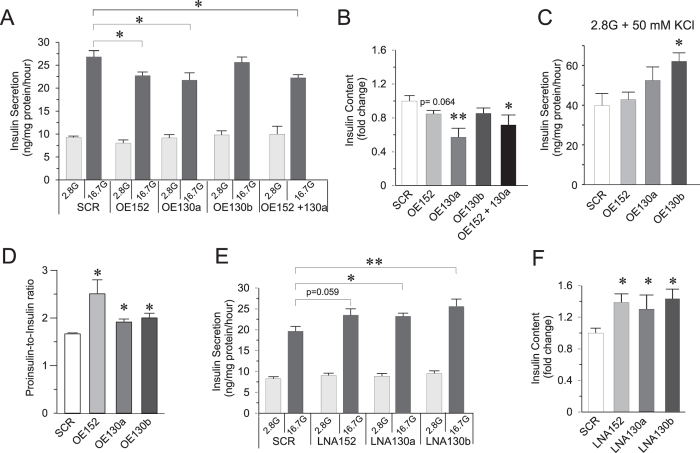
Effect of over-expression or knock-down of miRNAs on GSIS. **(A)** Insulin secretion measured upon over-expression (OE) of miRNAs (OE152, OE130a and OE130b) or combination of OE152 and OE130a in INS-1 832/13 cells. **(B)** Measurement of insulin content upon miRNA over-expression in INS-1 832/13 cells. **(C)** Insulin secretion of OE-miRNA cells at 2.8 G with 50 mM KCl. **(D)** Levels of pro-insulin to insulin ratio in OE-miRNA cells. **(E,F)** Insulin secretion, and content measurement upon knock-down of miRNAs using LNA (LNA152, LNA130a, LNA130b) in INS-1 832/13 cells, respectively. For all experiments, data are presented as average of n = 3–4 biological replicates each with 3–4 technical replicates. Data are mean ± SEM, *p < 0.05 and **p < 0.01 vs scramble using Student’s two-sided t-test.

OE152 and OE130a resulted in 15–20% reduced GSIS and up to ≈40% reduced insulin content compared to SCR (Fig. 2A,B). When miR-130a and miR-152 in combination were each overexpressed at half the amount of final concentration than when each miRNA was overexpressed separately, similar magnitude of reduction was observed for GSIS and insulin content, demonstrating the additive effect of miR-152 and miR-130a. Activation of insulin secretion independent of ATP by addition of 50 mM KCl in the presence of low levels of glucose did not show any reduction in insulin secretion upon miRNA overexpression (Fig. 2C). Interestingly, a significant increase in insulin secretion was seen in OE130b cells, although this increase was not significantly different when compared to OE130a. To determine whether the maturation of insulin is also affected by elevated miRNA levels, we measured the total proinsulin-to-insulin ratio in miRNA overexpressing cells. OE152 cells showed up to ≈50% increased proinsulin-to-insulin ratio, while OE130a and OE130b cells had ≈25% significant increased ratio (Fig. 2D). Taken together, these results suggest that exocytotic processes distal to plasma membrane depolarization are intact^27^,^28^ and that the main negative effect of the upregulated miRNAs under study is on insulin content levels, possibly influenced by upstream insulin maturation processes.

In contrast to the effect of miRNA-overexpressing beta cells, LNA152, LNA130a and LNA130b resulted in 20–30% increase in GSIS and up to ≈45% increased insulin content vs SCR control (Fig. 2E,F). These results further support the direct regulatory effect of the three miRNAs in stimulus-secretion coupling in the beta cells, and imply that insulin secretion may be positively influenced by reducing the levels of specific miRNAs.

### Identification of mRNA targets

To determine the putative targets through which the miRNAs under study may regulate insulin secretion in the beta cell, we performed bioinformatics analysis by using multiple target prediction algorithms as implemented in the web-server miRWalk2.0^29^. Previous reports regarding decreased glucokinase (*GCK*) mRNA expression in the pancreatic islets of T2D humans^30^ and GK rats^31^, and deficiency of pyruvate dehydrogenase activity^17^ in the pancreatic islets of diabetic GK rats led us to focus on the potential targeting of miR-130a/b and miR-152 within the 3′UTR and coding sequence (CDS) regions of *GCK/Gck* and *PDHA1/Pdha1* in human/rat (Supplementary Table 2).

**Figure 3:**
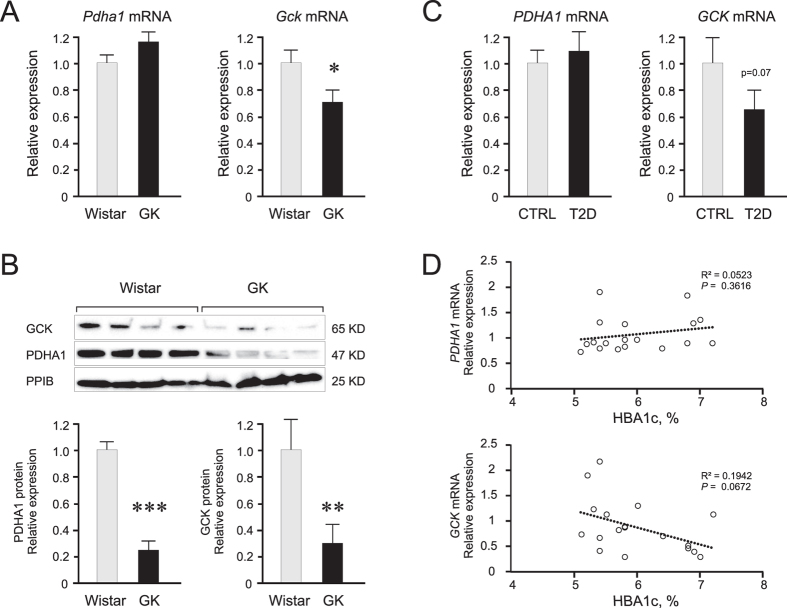
Expression of *Pdha1*/*Gck* in rat or *PDHA1/GCK* in human islets, and correlations to donor HbA1c levels. **(A)** Reduced mRNA levels of *Gck* in the GK (n = 11) islets compared to those of control Wistar (n = 4). **(B)** Reduced protein levels of both PDHA1 and GCK in the GK (n = 9–11) compared to Wistar (n = 4) islet preparations. Blots were cut around specified molecular weights prior to separate probing with specific antibodies. Unedited blots are shown in Supplementary Fig. S4A. **(C)** Reduced trend of *GCK* mRNA expression in the islets from T2D donors (n = 9) compared to controls (n = 10). **(D**) Negative trend of correlation between *GCK* mRNA expression and donor HbA1c levels (n = 18) using simple linear regression with F-test to determine significance at p < 0.05. For differential expression of mRNA or protein levels, Student’s t-test (unpaired, one-sided) was used to determine significance. Error bars where present are S.E.M. *p < 0.05, **p < 0.01, ***p < 0.001.

To lend support to the hypothesis that the three upregulated miRNAs via downregulation of putative targets, GCK and PDHA1, are contributing to perturbed functions of pancreatic islet cells in the T2D model GK rats and human T2D patients, we measured their expression by qPCR and/or western blot. In the GK rat islets, we observed downregulation of the *Gck* mRNA but no significant change in *Pdha1* mRNA (Fig. 3A). Nonetheless, the protein levels of both GCK and PDHA1 were considerably reduced (Fig. 3B). For the human islets, due to limited amount of sample, we could only measure mRNA levels by qPCR (Fig. 3C). We did not find significant differences in the mRNA expression of *PDHA1* or *GCK* in the islets of controls vs T2D donors, although we saw a clear trend of reduced *GCK* mRNA expression in T2D islets (p = 0.07) which agrees with previous findings regarding the reduced *GCK* mRNA in a larger cohort of T2D islets^30^. Notably in the human islets, we also demonstrated a trend of negative correlation between *GCK* mRNA expression and the long-term indicator of glycaemic control, HbA1c status of the donors (Fig. 3D).

In the rat islet samples, we clearly observed a larger differential regulation between GK and Wistar in the protein level (Fig. 3B) than in the mRNA level (Fig. 3A). This indicates that the miRNAs in this case are acting on the level of translational repression of target genes. As shown in the case of *Pdha1* in rat islets, the mRNA levels were not differentially-regulated between GK and Wistar, but the protein level was ≈75% lower in the GK rat islets. The *Pdha1* and *Gck* mRNA expression patterns exhibited in the rat islets between GK and Wistar (Fig. 3A) were strikingly similar with those exhibited in the human islets between control and T2D donors (Fig. 3C). We therefore hypothesize that the protein levels of PDHA1 and GCK will be similarly reduced in the human islets from T2D donors.

As a first indication of the miRNA-mRNA regulatory interactions in the beta cell, we observed reciprocal expression patterns of the miRNAs and their putative targets after just one-hour incubation of INS-1 832/13 cells in 2.8 mM (low) and 16.7 mM (high) glucose. The expression of all the three miRNAs were 60–80% lower in high glucose compared to low glucose (Supplementary Fig. S2A), whereas the corresponding GCK and PDHA1 targets showed ≈25% and ≈50% increased protein levels, respectively (Supplementary Fig. S2B).

**Figure 4:**
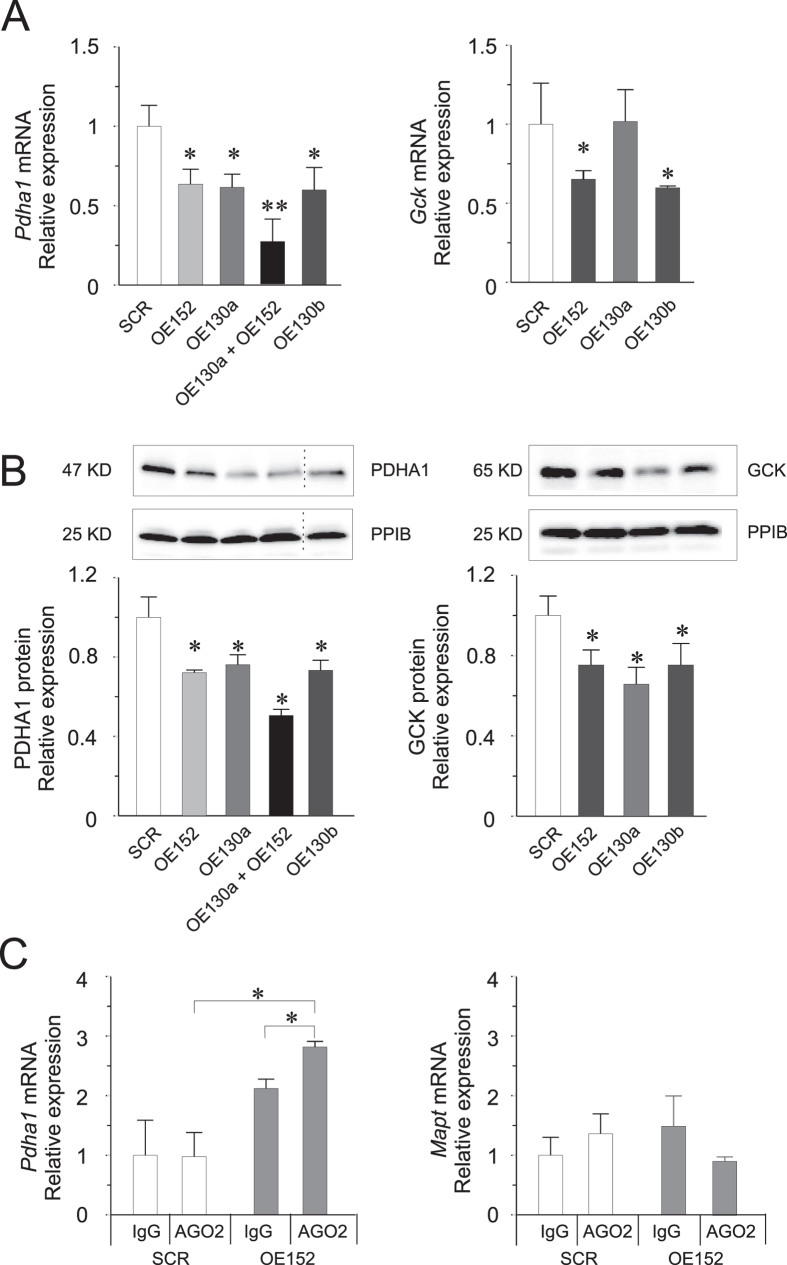
Validation of miRNA-target interactions. **(A)** Reduced mRNA levels of *Pdha1* and *Gck* upon miRNA overexpression. **(B)** Reduced protein levels of PDHA1 and GCK upon miRNA over-expression. OE130b lane was derived from a separate western blot run. Unedited blots are shown in Supplementary Fig. S4B. **(C)** Expression of 3′UTR target region of *Pdha1* or *Mapt* (non-specific control) after co-immunoprecipitation with anti-AGO2 in OE152 cells. Data are presented as mean ± SEM. n = 3–6, *p < 0.05 and **p < 0.01 vs SCR using Student’s two-sided test.

We then demonstrated negative regulatory effect of the miRNAs on the putative targets by over-expression of the miRNAs. We found that miR-130a, miR-130b and miR-152 or combination of miR-130a/miR-152 resulted in significant decrease in the expression of *Pdha1* both in the mRNA and protein levels (Fig. 4A,B). The combined miRNAs were each transfected at half the concentrations, compared to when transfected individually, and although their effect on the level of *Pdha1* expression seemed to be greater, the difference was not found to be statistically significant. For *Gck* targeting, we found reduced GCK protein by ≈35% and ≈25% in OE130a and OE130b cells, respectively (Fig. 4B). Additionally, we also observed reduced *Gck* expression both in the mRNA and protein levels, upon miR-152 overexpression (Fig. 4A,B).

To validate direct biochemical interactions between the miRNAs and target mRNAs, we utilized the anti-AGO2 RNA immunoprecipitation (RIP) assay, using anti-AGO2 to isolate both the miRNA and the interacting target mRNA 3′UTR fragment in INS-1 832/13 cells. We observed a significant increase in the levels of the interacting *Pdha1* 3′UTR in AGO2 RIP compared to the non-specific binding control IgG RIP in response to OE152 (Fig. 4C). However, we found no enrichment of the *Pdha1* 3′UTR fragment nor of the *Gck* 3′UTR fragment predicted to interact with miR-130a (Supplementary Fig. S3). These results provide strong evidence that at least miR-152 via AGO2 association, directly targets *Pdha1* resulting in both reduced transcript and protein expression levels.

### Over-expression of miRNAs decreased ATP content and cytosolic ATP:ADP ratios in INS-1 832/13 cells

**Figure 5:**
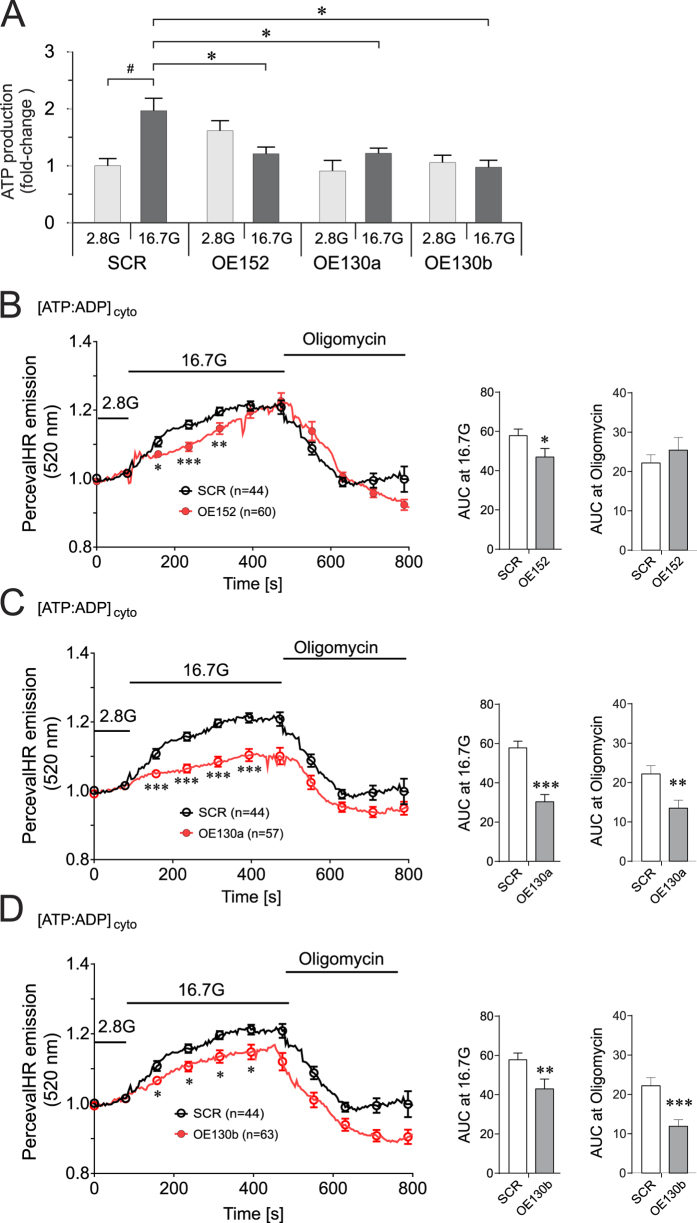
ATP production and cytosolic ATP dynamics in INS-1 832/13 cells upon miRNA-overexpression. **(A)** Luciferase-based luminescent detection of total ATP content in INS-1 832/13 upon over-expression of miRNAs. ATP produced at 2.8 mM glucose in SCR was used as calibrator. Data are presented as mean ± SEM. n = 3, *p < 0.05, one-way ANOVA with Dunnett’s post-hoc. **(B)** OE152**, (C),** OE130a, and **(D)** OE130b PercevalHR ATP:ADP ratio fluorescent reporter measurement in single-cells during GSIS, and oligomycin treatment. *p < 0.05, **p < 0.01, ***p < 0.001 using Mann-Whitney U test (unpaired, two-sided).

To resolve the net effect of the upregulated miRNAs on cellular energy metabolism in the beta cells, we analysed the oligomycin-sensitive ATP content by luciferase-based luminescent assay. Compared to controls, the cells overexpressing the miRNAs failed to increase their ATP levels upon incubation from 2.8 mM to 16.7 mM glucose, exhibiting substantial decrease, 40–50%, in ATP content at high glucose concentration (Fig. 5A).

A limitation of measuring ATP content in whole cell extracts is that total, and not metabolically regulated, ATP levels are determined. Furthermore, it is an end-point determination. Therefore, we utilized the genetically-encoded PercevalHR ATP sensor to assess the dynamics of ATP concentration changes as the ATP:ADP ratio in live single cells^20^. We observed profound qualitative differences in the trajectory of cytosolic ATP levels among the different miRNA-overexpressing (miRNA-OE) cells and controls during GSIS (Fig. 5B–D). We then calculated the area under the curve (AUC) during the period of high glucose stimulation, as well as the AUC in the presence of the ATP synthase blocker, oligomycin. In agreement with decreased ATP content in the cells at stimulatory glucose concentration upon miRNA overexpression, we observed reduced cytosolic ATP:ADP ratios in all miRNA-OE cells at 16.7 mM glucose (Fig. 5B–D).

Interestingly, while all the miRNA-OE cells displayed significantly lower ATP:ADP ratios at 16.7 mM glucose, the magnitude of decrease was smaller for OE152 cells. Moreover, as opposed to OE130a or OE130b cells, the decrease in ATP:ADP ratios for OE152 cells upon oligomycin treatment was not significantly reduced compared with the controls (AUC graphs Fig. 5B–D).

Oligomycin treatment effectively blocks ATP synthase within the mitochondria, forcing glycolysis to provide for cellular ATP production. Therefore, one might expect similar defect in cytosolic ATP/ADP ratio between OE152 and OE130a/b since all these miRNAs can negatively regulate the primary glycolytic enzyme, GCK. However, one may also argue that miR-130a/b can have other target enzymes within the glycolytic pathway which contribute to the further reduction of cytosolic ATP/ADP upon oligomycin-mediated inhibition of mitochondrial ATP synthase. We therefore performed more in-depth bioinformatics prediction for miR-130a binding to other glycolysis-related enzymes^32^ in the rat beta cell such as in phosphofructokinase (*Pfkm*), pyruvate kinase (*Pklr*), malic enzyme (*Me1*), ATP-citrate lyase (*Alcy*), and glyceraldehyde-3-phosphate dehydrogenase (*Gapdh*). However, using bioinformatics prediction we did not find any of these genes to be potentially targeted by miR-130a/b. Another line of reasoning is that miR-152 may target genes that require cytosolic ATP such that downregulation of these targets will result in eventual accumulation of cytosolic ATP concentration as observed in the OE152 cells. Indeed, when we performed gene ontology (GO) enrichment on predicted targets unique to miR-152, we saw significant enrichment for the gene ontology molecular function category called “ATP-binding” (Benjamini corrected p-value = 0.013). We did not see enrichment in ATP-binding category for genes containing only miR-130a/miR-130b putative binding sites (Supplementary Table 3). Among the 172 ATP-binding genes with unique putative miR-152 binding sites are the highly expressed adenylyl cyclase in the pancreatic islets, *Adcy5* and *Adcy6*, involved in the conversion of ATP to cAMP^33^ and two Na^+^/K^+^ ATPase transporting subunit genes (*Atp1a2* and *Atp1a3*) involved in establishing and maintaining the electrochemical gradients of Na^+^ and K^+^ ions across the plasma membrane. For this reason, we hypothesize that overexpression of miR-152, aside from targeting proteins required for ATP-generation such as *Pdha1* and *Gck*, they may also target genes that require ATP in fundamental cellular processes in the beta cell.

### Knock-down of PDHA1 in INS-1 832/13 reduces GSIS, ATP production and cytosolic ATP levels

**Figure 6:**
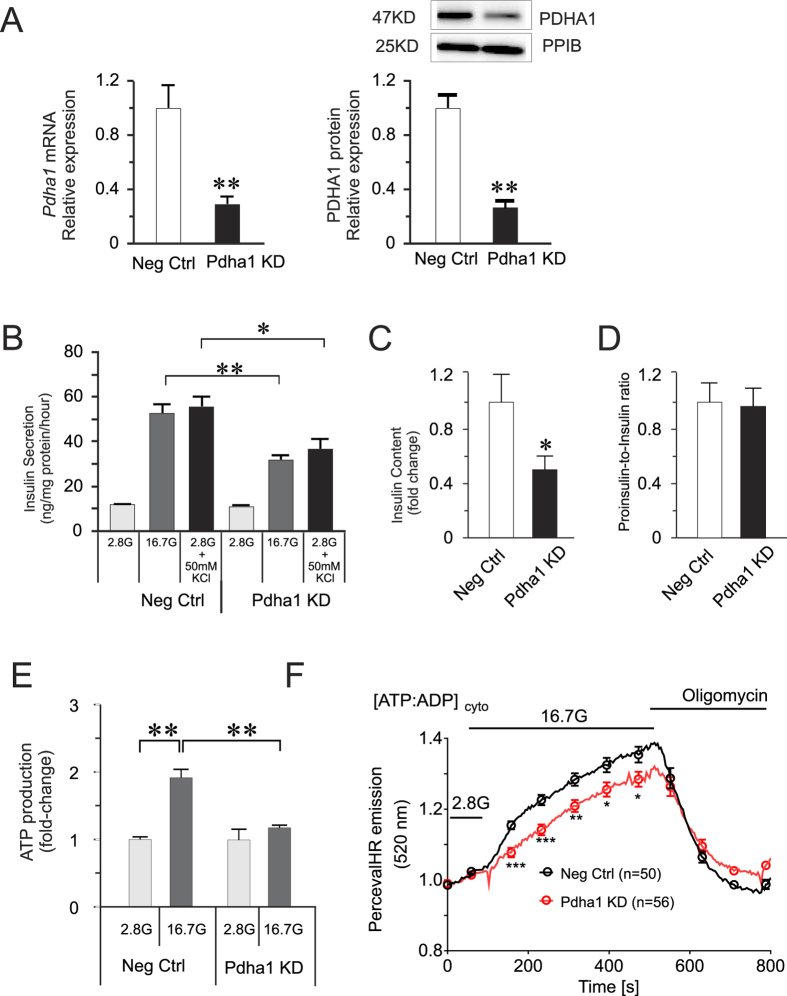
Effect of siRNA knock-down of PDHA1 on GSIS and ATP dynamics. **(A)** Expression of *Pdha1* in INS-1 832/13 cells after knock-down. Unedited blots are shown in Supplementary Fig. S4C. **(B)** Insulin secretion, **(C)** Insulin content and, **(D)** proinsulin-to-insulin ratio measurement after ≈70% knock-down of PDHA1 in INS-1 832/13. The data are presented as mean ± SEM of n = 3–5 biological replicates, *p < 0.05 and **p < 0.01 vs Neg Ctrl using Students two-tailed test or one-way ANOVA with Dunnett’s post-hoc for multiple groups. **(E)** ATP content upon PDHA1 knock-down. **(F)** PercevalHR ATP:ADP ratio fluorescent reporter measurement. *p < 0.05, **p < 0.01, ***p < 0.001 using Mann-Whitney U test (unpaired, two-sided).

Since miRNAs may have multiple targets, we wanted to confirm whether the effect of miRNA-mediated downregulation of PDHA1 on beta cells can be recapitulated by directly reducing PDHA1 levels by siRNA. We attained ≈70% knockdown of *Pdha1*, both in mRNA and protein levels, in INS-1 832/13 cells (Fig. 6A). This resulted in ≈40% reduced insulin secretion at both 16.7 mM glucose and at 50 mM KCl, and 50% reduction in insulin content (Fig. 6B–C). We saw reduced pro-insulin levels which resulted in no significant change in the proinsulin-to-insulin ratio in the PDHA1 KD cells (Fig. 6D). This indicates that the rate of insulin maturation is intact. The knock-down of *Pdha1* also resulted in reduced ATP content by ≈40% (Fig. 6E), and significantly lower levels of cytosolic ATP:ADP ratios during GSIS (Fig. 6F). To summarize, the similar beta cell functional deficiencies observed between the OE152 cells and PDHA1 KD cells, support the miR-152- mediated control of GSIS at the level of cellular metabolism via the negative regulation of PDHA1.

## Discussion

In this study, we showed that islets from hyperglycaemic human donors contain elevated levels of miR-130a, miR-130b and miR-152. We further validated the over-expression of the same miRNAs in the pancreatic islets of the T2D GK rat model. This suggests that similar pathophysiological processes leading to beta cell dysfunction, mediated by the dysregulated miRNAs, may be at play. The remarkable co-expression among the three miRNAs in human pancreatic islets also indicates a convergent transcriptional regulatory response to environmental stimuli.

The mechanism by which the three miRNAs in this study are upregulated in the pancreatic islets of GK rats and T2D humans is under investigation. Of note, the miR-152 and miR-130a are among the 24 miRNAs we previously showed to be upregulated in the pancreatic islets of GK rats^10^. We hypothesized that this was due to a perturbed transcriptional regulation and we therefore performed promoter analysis to find out DNA sequence motifs common to the upregulated miRNAs. However we only found common DNA sequence motif in the promoter region of two other upregulated miRNAs in the GK rat islets, miR-132 and miR-212^34^. In that study our group showed putative transcription factor binding sites for Calmodulin Binding Transcription Activator 1 (Camta1) and NK2 homeobox protein, Nkx2-2. Elsewhere, we and others also demonstrated cAMP-dependent regulation of the miR-132/212 cluster through a PKA-dependent mechanism^35^ involving cAMP-response element (CRE)-binding proteins and CRTC1^36^. In *db*/*db* and high-fat diet fed mice, miR-132 and miR-184 in the pancreatic islets were suggested to be induced by hyperglycaemic and hyperlipidaemic conditions typically encountered in prediabetic and diabetic states^37^. Concerning the miRNAs studied here, we previously showed that miR-130a expression is downregulated with increased glucose concentration in Wistar rat islets^10^, which was supported by our findings in INS-1 832/13 cells for miR-130a, miR-130b and miR-152 (Supplementary Fig. S2).

Most miRNA profiling studies done so far in relation to diabetes have been exploratory in nature and the exact mechanism of deregulated miRNA expression is largely unexplored. In one miRNA profiling study of human type-2 diabetes islets, a cluster of miRNAs in the so called DLK1-MEG3 locus at chromosome 14q32 was found to be imprinted and containing a promoter region that is hypermethylated leading to downregulation of a dozen miRNAs^38^. However, it was not established whether the aberrant hypermethylation was the cause or consequence of the disease state. Investigating the mechanisms causing dysregulated miRNA expression in pathophysiological conditions is therefore a topic of utmost importance.

Impaired GSIS is the hallmark phenotype of the GK rat, reflecting the pancreatic beta cell dysfunction in humans with T2D^11^. In our previous investigation of global miRNA expression in GK rat islets, we focused on the negative impact of the dysregulated miRNAs on the insulin exocytotic process, compatible with previous and recent findings on reduced exocytotic proteins in the pancreatic islets of the GK rats^13^,^14^, and in those of T2D donors^39^. Here, we instead elucidated the contribution of dysregulated miRNAs in beta cell energy metabolism. Prompted by previous findings regarding the deteriorating expression and/or dysfunction of key metabolic enzymes in the beta cell of GK rats and in the islets of T2D individuals, such as glucokinase^30^,^31^ and PDHA1^17^, we concentrated our target validation on these two genes which are among the many putative mRNA targets of the miRNAs under study. Consequently, we found reduced PDHA1 and GCK proteins in the GK islets, and a trend for reduced *GCK* mRNA expression in the islets of human T2D in agreement with previous findings^30^.

Subsequently, we could show negative regulatory effects of miR-130a and miR-130b on glucokinase and PDHA1, and the direct biochemical interaction of miR-152, with *Pdha1* mRNA using the AGO2 RIP assay. However, it has been found in mammalian cells that other non-catalytic AGO proteins such as AGO1 and AGO3 may also be equally loaded with distinct^40^ or random^41^ miRNAs bound to mRNA in the RNA-induced Silencing Complex (RISC) destined for target repression. It is therefore plausible that the direct repression of PDHA1 or GCK by miR-130a may have occurred via AGO1 or AGO3. Taken together, the negative effect of the over-expressed miRNAs on their targets involved in energy metabolism resulted in reduced cytosolic ATP levels, and ultimately in impaired GSIS in the pancreatic beta cell.

PDHA1 is a component of the mitochondrial holoenzyme pyruvate dehydrogenase complex (PDC) which is responsible for the conversion of pyruvate to acetyl-CoA and CO_2_, essentially linking glycolysis and the tricarboxylic acid (TCA) cycle. Robust insulin secretion in primary beta cells and insulin-secreting cell lines relies on the normal metabolic output emanating from the TCA cycle^32^. The catalytic components of PDC are known to be tightly-regulated, and maintaining stoichiometric amounts is essential for a functionally-active PDC^42^. Indeed, beta cell-specific deletion of PDHA1 alone in a murine model (β-PDHKO) led to a defect of GSIS both *in vitro* and *in vivo*, as well as reduced islet insulin content resulting in hypoinsulinemia and hyperglycemia in the animals^24^. Recent investigation on the β-PDHKO mice further revealed beta cell development and maturation defects, possibly due to deficient levels of several transcription factors with key roles in beta cell lineage commitment, such as PDX1, Neurogenin3, and NeuroD1^43^. Therefore, the pleiotropic effects of PDC on pancreatic beta cell development and function further highlight the role of glucose metabolism in the pathophysiology of impaired glucose homeostasis.

PDC-deficiency mainly caused by mutations in the *PDHA1* gene in humans are rare, and is mostly manifested as severe neuropathies leading to death at very young age^44^. Nonetheless, a report on a case of a child with PDH deficiency suffering from diabetes due to insufficient insulin^45^ should prompt further investigation on the potential co-morbidity of diabetes with the neuropathies in this genetic disease. Indeed, many commonalities between the central nervous system and human islet pathophysiological features have been noted in the literature, e.g. effects of neuronal cell adhesion molecules, EPHs/ephrins, in beta cell GSIS^46^.

We showed that the over-expression of the miRNAs under study, or the knockdown of the validated target, PDHA1, in a pancreatic beta cell line with robust insulin secretion capacity, resulted in changes in cellular bioenergetics, in which the net ATP output was substantially reduced. We could further resolve the ATP dynamics during GSIS in single cells using the PercevalHR reporter. The sensitivity of the PercevalHR system to physiological concentration range of ATP, reflecting the ATP:ADP ratio in the beta cells has previously been shown and found to reliably report changes in cellular metabolism^19^,^20^. This allowed us to see specific effects of the miRNAs on cytosolic ATP levels. For instance, we could observe that miR-152 impacted less on the ATP:ADP ratios during GSIS than either miR-130a or miR-130b. Moreover, the significant reduction of ATP levels after oligomycin treatment was only observed in miR-130a/b over-expressing cells. The similar ATP dynamics profile of miR-130a and miR-130b confirmed the similar regulatory targets of these miRNAs by virtue of their identical seed sequence.

The striking resemblance of ATP concentration changes during GSIS, and after inhibition of ATP synthase (oligomycin treatment) between OE152 cells and PDHA1 KD cells lend strong support to direct miR-152 mediated regulation of PDHA1. However, although both treatments resulted in impaired GSIS, the effect on insulin content was more pronounced when PDHA1 was directly silenced. These phenotypic discrepancies were due to expected differences in the magnitude of PDHA1 downregulation, i.e. miRNA-mediated control of PDHA1, was weaker than directly knocking down the levels of PDHA1 by siRNA. Additionally, the multiple targeting of miRNA may also lead to activation/inactivation of pathways influencing other beta cell functions which may not be sensitive to silencing a single target gene.

The transient modulation of miR-130a, miR-130b, or miR-152 in INS-1 832/13 cells by either over-expression or knock-down resulted in reciprocal effects on GSIS, but not KCl-induced insulin secretion agrees with targets primarily involved in modulating the cytosolic ATP concentration. Interestingly, the results on insulin secretion were related to changes in both insulin content, and proinsulin-to-insulin ratio. The biosynthesis and processing of mature insulin, and its subsequent packaging into granules destined for secretion are known to be ATP-requiring processes^22^,^23^. More important clinically, elevated proinsulin levels have previously been recognized as indicative of impaired beta cell secretory capacity in non-insulin-dependent diabetes mellitus^47^.

A notable result in this study was the further improvement in both insulin secretion and insulin content upon LNA anti-miR treatment of the beta cells. These findings have considerable implication in the development of RNA-based novel therapeutics, which target miRNAs in the diseased beta cell. In this aspect, combinatorial modulation of multiple dysregulated miRNAs could be a more efficacious path in rectifying diseased states than just modulating levels of a single miRNA. Moreover, LNA anti-miRs could most likely be used in combination, if not replace certain drugs currently used to improve insulin secretion *e.g.* sulfonylureas. These compounds have long been utilized to improve insulin secretion through binding to the sulfonylurea subunit SUR1 of the ATP-dependent K^+^ channel (K_ATP_ channel) affecting beta cell membrane potential^48^. Hypothetically, LNAs that knock down miR-130a/miR-130b/miR-152 would affect the glucose conversion pathway in the mitochondria and assist in stabilizing the intracellular ATP to an optimal level. ATP acts on the Kir6.2 subunit of the ATP-dependent K^+^ channel and increased ATP leads to closure of the channel and membrane depolarization^48^. ATP could also directly amplify exocytosis of insulin granules^49^. Hence, this anti-miR treatment would most likely work in concert with sulfonylureas to improve control of insulin output from the beta cell.

**Figure 7:**
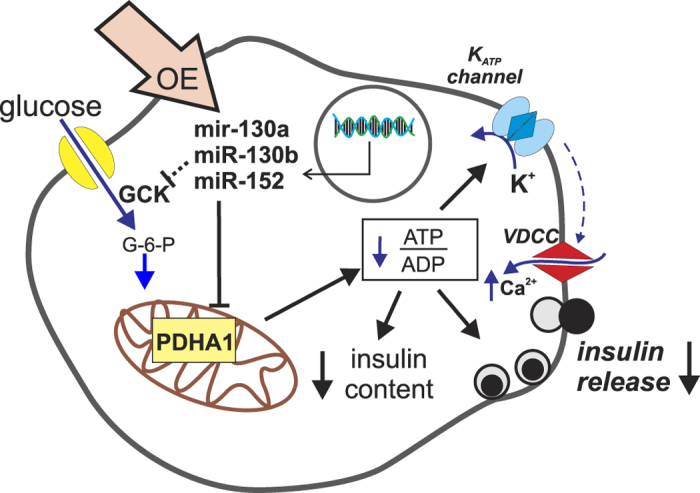
Model of miRNA impact on GSIS via regulation of genes involved in ATP production. In INS-1 832/13 cells, normal levels of miRNAs 130a/b and 152 has no detrimental effect on PDHA1 or GCK expression resulting in robust GSIS. Upon up-regulation of the miRNAs, PDHA1 and GCK levels are reduced contributing to profound reduction in cytosolic ATP:ADP ratio, which ultimately lead to both reduced insulin content and defective insulin release.

To conclude, we could show that miR-130a, miR-130b and miR-152 influence the metabolic control of GSIS via modulation of ATP levels, partially through targeting of PDHA1 and GCK in the pancreatic beta cell (Fig. 7). Modulating the expression of miRNAs to improve beta cell function in T2D is a promising approach but using this novel therapeutic tool requires further studies.

## Methods

### Ethical statement

For experiments involving human pancreatic islets, all procedures were approved by Uppsala and Lund University Ethics committees, and fully-complied with the guidelines and regulations as stated in the ethical permit, Dnr 2011/263 issued to Lund University Diabetes Centre (LUDC) and Excellence in Diabetes Research in Sweden (EXODIAB) regarding use of donated human tissues.

Experiments on rodents were performed in full-compliance of guidelines and regulations as stated in the ethical permit, M 105-15 issued by Malmö/Lund Ethical Committee on Animal Research.

### Reagents

Unless otherwise stated, all chemicals were from Sigma Aldrich (MO, USA).

### Human pancreatic islets

Human pancreatic islets from cadaver donors were procured from the Human Tissue Lab EXODIAB/LUDC through the Nordic Network for Islet Transplantation (http://www.nordicislets.org). Upon receipt, the islets were handpicked under stereomicroscope. The donors were grouped according to Fadista *et al*. based on the glycated haemoglobin (% HbA1c) levels^50^. Here we used pancreatic islets from normal glucose tolerant (NGT) donors (HbA1c < 6%; *n* = 20), impaired glucose-tolerant donors (IGT) (6% ≤ HbA1c < 6.5%; *n* = 11), and T2D donors (HbA1c ≥ 6.5%; *n* = 11) (Supplementary Table 1).

### Rat pancreatic islets

Pancreatic islets were isolated from male Goto-Kakizaki and control Wistar rats as previously described^10^. All animals were kept in standard 12-hour (h) light-dark cycle and were given standard chow and water *ad libitum*. The animals were used at 8–15 weeks of age, at which point the non-fasting blood glucose of GK rats (22.3 ± 1.2 mmol/L, N = 11) was significantly higher than those of Wistar controls (6.3 ± 0.2 mmol/L, N = 4) (Student’s t-test, two-sided, p < 0.001).

### Cell Culture

Rat INS-1 832/13 cell line^51^ was maintained in a complete RPMI 1640 medium with 11.1 mM D-glucose supplemented with 10% Fetal Bovine Serum (FBS), 2% INS-1 supplement, 5 mL Penicillin/Streptomycin (10000 U/10 mg/mL) and 10 mM Hepes (HyClone, UT, USA). Cells were incubated in a humidified atmosphere with 5% CO_2_ at 37 °C.

### Over-expression and knock-down of miRNAs

INS-1 832/13 cells were seeded (300,000 cells per well) in a 24-well plate with 1 mL/well complete RPMI 1640 medium without antibiotics a day before transfection. Cells were transfected with mature miRNAs called PremiR™ miRNA Precursor from Life Technologies (CA, USA): PremiR-Scramble (AM17110), Premir-130a (PM105106), Premir-130b (PM10777), Premir-152 (PM12269), or miRcury LNA miRNA inhibitors from Exiqon (Denmark): LNA Scramble (#199005-00), LNA130a (#4102212-001), LNA130b (#4102260-001) and LNA152 (#4103524-001) or after reaching ≈60% confluence using Lipofectamine RNAiMAX. A final transfection volume of 600 μL per well contained 50 nM of Pre-miR or LNA in Opti-MEM reduced serum media and 1.5 μL of Lipofectamine RNAiMAX (Life Technologies, CA, USA). In cells transfected with combined Pre-miR-152 and Pre-miR-130a, the concentration of each Pre-miR was reduced to 25 nM. After 6 h transfection, 500 μL of complete RPMI 1640 medium without antibiotics were added to each well. Medium was changed to complete RPM1 1640 with antibiotics after 24 hours of transfection. Cells were assayed for insulin secretion after reaching ≈100% confluence, while protein and RNA samples were extracted from replicate wells at the same time, 72 h post-transfection.

### siRNA knock-down of Pdha1

Using the same transfection protocol as when we overexpressed or knocked-down the miRNAs, we knocked-down Pdha1 in INS-1 832/13 cells in a 24-well plate using Silencer^®^ Select Pre-Designed siPdha1 (#s131695, Life Technologies, CA, USA). Cells were assayed for insulin secretion after reaching ≈100% confluence, while protein and RNA samples were extracted from replicate wells at the same time, 72 h post-transfection.

### Insulin Secretion Assay

Confluent plates of INS-1 832/13 cell lines were washed twice carefully with 1 mL pre-warmed Secretion Assay Buffer (SAB), pH 7.2 (1.16 mM MgSO_4,_ 4.7 mM KCl, 1.2 mM KH_2_ PO_4_, 114 mM NaCl, 2.5 mM CaCl_2_, 25.5 mM NaHCO_3_, 20 mM HEPES and 0.2% Bovine Serum Albumin) containing 2.8 mM glucose. Cells were then pre-incubated in fresh 2 mL SAB with 2.8 mM glucose for 2 h. The cells were stimulated for 1 h in 1 mL SAB with 2.8 mM glucose or 16.7 mM glucose or 2.8 mM glucose with 50 mM KCl at 37  °C. Insulin levels were measured using Coat-A-Count radioimmunoassay kit, according to the manufacturer’s instructions (Millipore Corporation, MA, USA) and the values were normalized to total protein from each well. Total protein from each well was extracted by using 200 μL RIPA buffer: 0.1% SDS, 150 nM NaCl, 1% Triton X-100, 50 mM Tris-Cl, pH 8 and EDTA-free protease inhibitor (Roche, NJ, USA). The protein content was analyzed by BCA assay (Pierce, IL, USA) on Bio-Rad Model 6870 microplate reader.

### Total insulin and Proinsulin measurement

Cells transfected with mature miRNAs or siRNA as described above were lysed in 200 μL RIPA buffer after 72 h post-transfection. Total insulin and proinsulin were determined using Mercodia High Range Rat Insulin ELISA and Rat/Mouse Proinsulin ELISA (Mercodia AB, Sweden) respectively according to the manufacturer’s protocol.

### RNA Extraction and quality control

Total RNA from INS-1 832/13 cells, GK rat and human islets was extracted by the Qiagen miRNeasy isolation kit according to the manufacturer’s recommendations (Qiagen, Hilden, Germany). The concentration of RNA was measured on a Nanodrop (ND-1000) spectrophotometer. The quality and integrity of RNA were evaluated by both spectrophotometry and electropherogram profiles using Nanodrop (ND-1000) and Experion’s automated electrophoresis system (Bio-Rad, CA, USA), respectively.

### Quantification of miRNAs and mRNAs by real-time quantitative PCR (qPCR)

cDNA was generated by using High Capacity cDNA Reverse Transcription kit according to the manufacturer’s instructions (Applied Biosystems, CA, USA). qPCR was performed in triplicates on 384-well plate using Applied Biosystems 7900HT standard RT-PCR system under default cycling parameters. Specific primers and probes from TaqMan^®^ MiRNA Assays (Applied Biosystems, CA, USA) were used to measure the expression levels of miR-130a-3p (#TM_000454), miR-130b-3p (#TM_000456), miR-152-3p (#TM_ 000475) and mRNA expression of their targets: Rat Pdha1 (Rn01424346_m1), Rat Gck (Rn00561265_m1), Human PDHA1 (Hs01049345_g1), and Human GCK (Hs01564555_m1). We used these snRNAs: U6 (#TM_ 001973) and U87 (#TM_ 001712) as endogenous controls for rat miRNA quantification or RNU48 (#TM_001006) for human miRNAs, while Rat Hprt1 (Rn_01527840_ml) and Rat Ppia (Rn_00690933_ml) or Human B2M (433766) and Human HPRT (4333768 F) were used for normalizing mRNA expression. All Taqman assays and qPCR reagents were purchased from Thermo Fisher. Relative quantification with multiple controls as applicable was done using the ΔΔC_t_ method.

### Validation of miRNA target using Anti-AGO2 RNA Immunoprecipitation (RIP)

Sigma’s Imprint^®^ RNA Immunoprecipitation Kit was used. RIP lysates from INS-1 832/13 cells (≈3 million cells per RIP) were immunoprecipitated with 2.5 μg of either rabbit IgG (I5006) or Anti-AGO2 antibody produced in rabbit (SAB4301150) according to the manufacturer’s protocol. RT-qPCR analysis of RNAs isolated from RIP was performed using SYBR^®^ Green JumpStart™ Taq ReadyMix™ (S4438) and the following 3’ UTR primers to detect mRNA of either Pdha1 or Mapt (non-target negative control): miR-152/*Pdha1* forward primer: 5′-TGT ATT CGA GGC TGG ACT CT-3′, miR-152/*Pdha1* reverse primer: 5′-ACA TAA CGG TCA GTG CCA AA-3′, miR-130ab/*Pdha1* forward primer: 5′-CGA ACA AGG GTC TTT CTG TGT A-3′, miR-130ab/*Pdha1* reverse primer: 5′-CAC ACA CAA ATC CTG CGT TTA C-3′, miR-130ab/*G*ck forward primer: 5′-CTT GCT AGA ATC AAC TAC AGA AA-3′, miR-130ab*/Gck* reverse primer: 5′-GGA AGC AAG AAT CGT GAA AG-3′, *Mapt* forward primer: 5′-TCT GTG AAT GTC CAT ATA GTG TAC TG-3′ and *Mapt* reverse primer: 5′-CAA CAG TCA GTG TAA ATC GTT TGT-3′.

### Western Blot Analysis

Total protein (10 μg/mL) extracted 72 h post-transfection was separated by 4–15% Mini-Protean TGX Precast gel from Bio-Rad Laboratories at 80 V. Protein was transferred to PVDF membrane, then blocked with 5% milk and 1% BSA in buffer consisting of 150 mM NaCl, 20 mM Tris -HCl, pH 7.5 and 0.1% (v/w) Tween for 1 h. The blot was probed with PDHA1 (1:500; # ab92696, Abcam, UK), GCK (1:500; #ab37796, Abcam, UK), or Cyclophilin B (1:2000; # ab16045 Abcam, UK) antibodies, and incubated overnight at 4 °C. Horseradish peroxidase conjugated goat anti-rabbit IgG, HRP-linked antibody (1:10 000; #7074; Cell Signaling Technology, Danvers, MA, USA) was used to detect the primary antibodies. Super Signal West Pico Chemiluminescent Substrate or Super Signal West Femto Maximum Sensitivity Substrate (Thermo Scientific, MA, USA) and AlphaImager (ProteinSimple, CA, USA) was used to detect protein and quantification was done by using FluorChem SP software (Protein Simple, CA, USA).

### ATP measurement

Luciferase-based luminescent assay (Bio Thema AB, Handen, Sweden) was used to measure total ATP levels after transfecting INS-1 832/13 with mature miRNA mimics or siRNA. Cells were pre-incubated in 2 mL SAB with 2.8 mM glucose for 2 h and stimulated with 1 mL SAB with 16.7 mM glucose for 30 min. The cells were suspended in 100 μL of lysis buffer containing 50 mM Tris (pH 7.5), 2 mM EDTA, 200 mM NaCl and 1% Triton X-100. The cells were quenched on dry ice for 15 min, thawed and sonicated for 5 seconds. An aliquot was diluted 1:50 in a reagent buffer consisting of 187.5 mM sucrose, 18.75 mM KH_2_PO_2_, 2.5 mM (CH_3_COO)_2_ Mg.4H_2_O and 0.625 mM EDTA, pH 7.0. A total volume of 100 μl of diluted sample was incubated with 25 μl of ATP monitoring reagent (Bio Thema AB, Handen, Sweden) at 25  °C for 5 min, and then ATP production was measured. Mitochondrial ATP synthase was inhibited by adding 0.6 mg/mL oligomycin as previously reported^32^, and any further ATP produced was measured. The amount of ATP produced during the measurements was calibrated by adding 2 μl of ATP standard diluted 5-fold in Tris-EDTA Buffer (Bio Thema AB, Handen, Sweden). Mitochondrial ATP production was calculated as difference between ATP produced before and after adding oligomycin. We used TECAN Infinite M200 with Magellan software for the ATP luminescence measurements.

### ATP measurement in single cells

Approximately 50,000 cells were seeded on poly-D-lysine (1 mg/mL) coated Lab-Tek chambered cover glass (Thermo Scientific, NY, USA). INS-1 832/13 cells were transfected with either Pre-miRs or siRNA together with 1 μg of PercevalHR plasmid DNA (Addgene ID: #21737)^18^ per well using Lipofectamine 3000 (Invitrogen) for a period of 48 h. The cells were pre-incubated in experimental buffer (pH 7.4): 3.6 mM KCl, 1.3 mM CaCl_2_, 0.5 mM MgSO_4_, 0.5 mM Na_2_HPO_4_, 10 mM Hepes, 5 mM NaHCO_3_ and 135 mM NaCl supplemented with 2.8 mM glucose for 90 min at 37  °C. Cover glass with adhered cells was mounted on the stage of Microscope (Zeiss Axiovert 200 M, Carl Zeiss AB, Stockholm, Sweden) equipped with confocal unit. PercevalHR was excited with laser light at 488 nm and emission was detected at 520 nm.

### Bioinformatics Analysis

We used the comprehensive atlas of predicted and validated miRNA-target interactions web server miRWalk 2.0 (http://zmf.umm.uni-heidelberg.de/apps/zmf/mirwalk2/generetsys-self.html)^29^ to identify putative targets of miR-130a-3p, miR-130b-3p, and miR-152-3p. To retrieve the predicted binding sites in the 3′UTR of *Pdha1* and *Gck* for AGO2-RIP assay, the sequences at TargetScan v7.1 for rat 3′UTRs (http://www.targetscan.org/vert_71/)^52^ was used.

To determine enriched Gene Ontology categories for genes with unique putative binding sites for either miR-130a/b or miR-152, we used the Database for Annotation, Visualization and Integrated Discovery (DAVID) v6.7 web server (https://david-d.ncifcrf.gov/)^53^.

### Statistical Analysis

Significant differences between two groups were determined using Student’s t-test. For multiple groups, between scramble and miRNAs in insulin secretion, insulin content and ATP measurements, significant differences were tested using one-way ANOVA followed by Dunnett’s multiple comparison test. The human islet qPCR data and AUCs derived from PercevalHR ATP:ADP curves were not normally distributed (Shapiro-Wilk Test) so differences were compared with Mann-Whitney U-test. All statistical tests were performed in IBM SPSS v22. Data were presented as mean ± SEM. Detailed statistical test parameters are indicated in the figure legend for each result.

## Additional Information

**How to cite this article:** Ofori, J. K. *et al*. Elevated miR-130a/miR130b/miR-152 expression reduces intracellular ATP levels in the pancreatic beta cell. *Sci. Rep.* **7**, 44986; doi: 10.1038/srep44986 (2017).

**Publisher's note:** Springer Nature remains neutral with regard to jurisdictional claims in published maps and institutional affiliations.

## Supplementary Material

Supplementary Information

## References

[b1] Ashcroft F. M. & Rorsman P. Diabetes mellitus and the beta cell: the last ten years. Cell 148, 1160–1171, doi: 10.1016/j.cell.2012.02.010 (2012). 22424227 PMC5890906

[b2] Wiederkehr A. & Wollheim C. B. Mitochondrial signals drive insulin secretion in the pancreatic beta-cell. Mol Cell Endocrinol 353, 128–137, doi: 10.1016/j.mce.2011.07.016 (2012). 21784130

[b3] Gerich J. E. Is reduced first-phase insulin release the earliest detectable abnormality in individuals destined to develop type 2 diabetes? Diabetes 51 Suppl 1, S117–121 (2002). 11815469 10.2337/diabetes.51.2007.s117

[b4] Prasad R. B. & Groop L. Genetics of type 2 diabetes-pitfalls and possibilities. Genes (Basel) 6, 87–123, doi: 10.3390/genes6010087 (2015). 25774817 PMC4377835

[b5] Eliasson L. & Esguerra J. L. Role of non-coding RNAs in pancreatic beta-cell development and physiology. Acta Physiol (Oxf) 211, 273–284, doi: 10.1111/apha.12285 (2014). 24666639

[b6] Esguerra J. L. & Eliasson L. Functional implications of long non-coding RNAs in the pancreatic islets of Langerhans. Front Genet 5, 209, doi: 10.3389/fgene.2014.00209 (2014). 25071836 PMC4083688

[b7] Bartel D. P. MicroRNAs: target recognition and regulatory functions. Cell 136, 215–233, doi: 10.1016/j.cell.2009.01.002 (2009). 19167326 PMC3794896

[b8] Pullen T. J., da Silva Xavier G., Kelsey G. & Rutter G. A. miR-29a and miR-29b contribute to pancreatic beta-cell-specific silencing of monocarboxylate transporter 1 (Mct1). Mol Cell Biol 31, 3182–3194, doi: 10.1128/MCB.01433-10 (2011). 21646425 PMC3147603

[b9] Thorrez L. . Tissue-specific disallowance of housekeeping genes: the other face of cell differentiation. Genome Res 21, 95–105, doi: 10.1101/gr.109173.110 (2011). 21088282 PMC3012930

[b10] Esguerra J. L., Bolmeson C., Cilio C. M. & Eliasson L. Differential glucose-regulation of microRNAs in pancreatic islets of non-obese type 2 diabetes model Goto-Kakizaki rat. PLoS One 6, e18613, doi: 10.1371/journal.pone.0018613 (2011). 21490936 PMC3072418

[b11] Portha B. . The GK rat beta-cell: a prototype for the diseased human beta-cell in type 2 diabetes? Mol Cell Endocrinol 297, 73–85, doi: 10.1016/j.mce.2008.06.013 (2009). 18640239

[b12] Galli J. . Genetic analysis of non-insulin dependent diabetes mellitus in the GK rat. Nat Genet 12, 31–37, doi: 10.1038/ng0196-31 (1996). 8528247

[b13] Nagamatsu S. . Decreased expression of t-SNARE, syntaxin 1, and SNAP-25 in pancreatic beta-cells is involved in impaired insulin secretion from diabetic GK rat islets: restoration of decreased t-SNARE proteins improves impaired insulin secretion. Diabetes 48, 2367–2373 (1999). 10580425 10.2337/diabetes.48.12.2367

[b14] Gaisano H. Y., Ostenson C. G., Sheu L., Wheeler M. B. & Efendic S. Abnormal expression of pancreatic islet exocytotic soluble N-ethylmaleimide-sensitive factor attachment protein receptors in Goto-Kakizaki rats is partially restored by phlorizin treatment and accentuated by high glucose treatment. Endocrinology 143, 4218–4226, doi: 10.1210/en.2002-220237 (2002). 12399415

[b15] Rosengren A. H. . Overexpression of alpha2A-adrenergic receptors contributes to type 2 diabetes. Science 327, 217–220, doi: 10.1126/science.1176827 (2010). 19965390

[b16] Granhall C., Rosengren A. H., Renstrom E. & Luthman H. Separately inherited defects in insulin exocytosis and beta-cell glucose metabolism contribute to type 2 diabetes. Diabetes 55, 3494–3500, doi: 10.2337/db06-0796 (2006). 17130497

[b17] Zhou Y. P., Ostenson C. G., Ling Z. C. & Grill V. Deficiency of pyruvate dehydrogenase activity in pancreatic islets of diabetic GK rats. Endocrinology 136, 3546–3551, doi: 10.1210/endo.136.8.7628391 (1995). 7628391

[b18] Berg J., Hung Y. P. & Yellen G. A genetically encoded fluorescent reporter of ATP:ADP ratio. Nat Methods 6, 161–166, doi: 10.1038/nmeth.1288 (2009). 19122669 PMC2633436

[b19] Li J., Shuai H. Y., Gylfe E. & Tengholm A. Oscillations of sub-membrane ATP in glucose-stimulated beta cells depend on negative feedback from Ca(2+). Diabetologia 56, 1577–1586, doi: 10.1007/s00125-013-2894-0 (2013). 23536115 PMC3671113

[b20] Tantama M., Martinez-Francois J. R., Mongeon R. & Yellen G. Imaging energy status in live cells with a fluorescent biosensor of the intracellular ATP-to-ADP ratio. Nat Commun 4, 2550, doi: 10.1038/ncomms3550 (2013). 24096541 PMC3852917

[b21] Schuit F. C., Huypens P., Heimberg H. & Pipeleers D. G. Glucose sensing in pancreatic beta-cells: a model for the study of other glucose-regulated cells in gut, pancreas, and hypothalamus. Diabetes 50, 1–11 (2001). 10.2337/diabetes.50.1.1 11147773

[b22] Rhodes C. J., Lucas C. A., Mutkoski R. L., Orci L. & Halban P. A. Stimulation by ATP of proinsulin to insulin conversion in isolated rat pancreatic islet secretory granules. Association with the ATP-dependent proton pump. J Biol Chem 262, 10712–10717 (1987). 2440873

[b23] Orci L. . Proteolytic maturation of insulin is a post-Golgi event which occurs in acidifying clathrin-coated secretory vesicles. Cell 49, 865–868 (1987). 3555846 10.1016/0092-8674(87)90624-6

[b24] Srinivasan M. . Beta-Cell-specific pyruvate dehydrogenase deficiency impairs glucose-stimulated insulin secretion. Am J Physiol Endocrinol Metab 299, E910–917, doi: 10.1152/ajpendo.00339.2010 (2010). 20841503 PMC3006256

[b25] Velho G. . Primary pancreatic beta-cell secretory defect caused by mutations in glucokinase gene in kindreds of maturity onset diabetes of the young. Lancet 340, 444–448 (1992). 1354782 10.1016/0140-6736(92)91768-4

[b26] Njolstad P. R. . Neonatal diabetes mellitus due to complete glucokinase deficiency. N Engl J Med 344, 1588–1592, doi: 10.1056/NEJM200105243442104 (2001). 11372010

[b27] Eliasson L. . Novel aspects of the molecular mechanisms controlling insulin secretion. J Physiol 586, 3313–3324, doi: 10.1113/jphysiol.2008.155317 (2008). 18511483 PMC2538808

[b28] Olofsson C. S. . Fast insulin secretion reflects exocytosis of docked granules in mouse pancreatic B-cells. Pflugers Arch 444, 43–51, doi: 10.1007/s00424-002-0781-5 (2002). 11976915

[b29] Dweep H. & Gretz N. miRWalk2.0: a comprehensive atlas of microRNA-target interactions. Nat Methods 12, 697, doi: 10.1038/nmeth.3485 (2015). 26226356

[b30] Del Guerra S. . Functional and molecular defects of pancreatic islets in human type 2 diabetes. Diabetes 54, 727–735 (2005). 15734849 10.2337/diabetes.54.3.727

[b31] Frese T., Bazwinsky I., Muhlbauer E. & Peschke E. Circadian and age-dependent expression patterns of GLUT2 and glucokinase in the pancreatic beta-cell of diabetic and nondiabetic rats. Horm Metab Res 39, 567–574, doi: 10.1055/s-2007-984471 (2007). 17712721

[b32] Malmgren S. . Tight coupling between glucose and mitochondrial metabolism in clonal beta-cells is required for robust insulin secretion. J Biol Chem 284, 32395–32404, doi: 10.1074/jbc.M109.026708 (2009). 19797055 PMC2781654

[b33] Tengholm A. Cyclic AMP dynamics in the pancreatic beta-cell. Ups J Med Sci 117, 355–369, doi: 10.3109/03009734.2012.724732 (2012). 22970724 PMC3497220

[b34] Mollet I. G., Malm H. A., Wendt A., Orho-Melander M. & Eliasson L. Integrator of Stress Responses Calmodulin Binding Transcription Activator 1 (Camta1) Regulates miR-212/miR-132 Expression and Insulin Secretion. J Biol Chem 291, 18440–18452, doi: 10.1074/jbc.M116.716860 (2016). 27402838 PMC5000089

[b35] Shang J. . Induction of miR-132 and miR-212 Expression by Glucagon-Like Peptide 1 (GLP-1) in Rodent and Human Pancreatic beta-Cells. Mol Endocrinol 29, 1243–1253, doi: 10.1210/me.2014-1335 (2015). 26218441 PMC4552436

[b36] Malm H. A. . Transcriptional regulation of the miR-212/miR-132 cluster in insulin-secreting beta-cells by cAMP-regulated transcriptional co-activator 1 and salt-inducible kinases. Mol Cell Endocrinol 424, 23–33, doi: 10.1016/j.mce.2016.01.010 (2016). 26797246

[b37] Nesca V. . Identification of particular groups of microRNAs that positively or negatively impact on beta cell function in obese models of type 2 diabetes. Diabetologia 56, 2203–2212, doi: 10.1007/s00125-013-2993-y (2013). 23842730

[b38] Kameswaran V. . Epigenetic regulation of the DLK1-MEG3 microRNA cluster in human type 2 diabetic islets. Cell Metab 19, 135–145, doi: 10.1016/j.cmet.2013.11.016 (2014). 24374217 PMC3932527

[b39] Andersson S. A. . Reduced insulin secretion correlates with decreased expression of exocytotic genes in pancreatic islets from patients with type 2 diabetes. Mol Cell Endocrinol 364, 36–45, doi: 10.1016/j.mce.2012.08.009 (2012). 22939844

[b40] Burroughs A. M. . Deep-sequencing of human Argonaute-associated small RNAs provides insight into miRNA sorting and reveals Argonaute association with RNA fragments of diverse origin. RNA Biol 8, 158–177 (2011). 21282978 10.4161/rna.8.1.14300 PMC3127082

[b41] Dueck A., Ziegler C., Eichner A., Berezikov E. & Meister G. microRNAs associated with the different human Argonaute proteins. Nucleic Acids Res 40, 9850–9862, doi: 10.1093/nar/gks705 (2012). 22844086 PMC3479175

[b42] Cullingford T. E., Clark J. B. & Phillips I. R. The pyruvate dehydrogenase complex: cloning of the rat somatic E1 alpha subunit and its coordinate expression with the mRNAs for the E1 beta, E2, and E3 catalytic subunits in developing rat brain. J Neurochem 62, 1682–1690 (1994). 8158120 10.1046/j.1471-4159.1994.62051682.x

[b43] Patel M. S., Srinivasan M., Strutt B., Mahmood S. & Hill D. J. Featured Article: Beta cell specific pyruvate dehydrogenase alpha gene deletion results in a reduced islet number and beta-cell mass postnatally. Exp Biol Med (Maywood) 239, 975–985, doi: 10.1177/1535370214531895 (2014). 24845368

[b44] Patel K. P., O’Brien T. W., Subramony S. H., Shuster J. & Stacpoole P. W. The spectrum of pyruvate dehydrogenase complex deficiency: clinical, biochemical and genetic features in 371 patients. Mol Genet Metab 105, 34–43, doi: 10.1016/j.ymgme.2011.09.032 (2012). 22079328 PMC3754811

[b45] Henwood M. J., Thornton P. S., Preis C. M., Chee C. & Grimberg A. Reconciling diabetes management and the ketogenic diet in a child with pyruvate dehydrogenase deficiency. J Child Neurol 21, 436–439 (2006). 16901455 10.1177/08830738060210051001

[b46] Konstantinova I. . EphA-Ephrin-A-mediated beta cell communication regulates insulin secretion from pancreatic islets. Cell 129, 359–370, doi: 10.1016/j.cell.2007.02.044 (2007). 17448994

[b47] Roder M. E., Porte D. Jr., Schwartz R. S. & Kahn S. E. Disproportionately elevated proinsulin levels reflect the degree of impaired B cell secretory capacity in patients with noninsulin-dependent diabetes mellitus. J Clin Endocrinol Metab 83, 604–608, doi: 10.1210/jcem.83.2.4544 (1998). 9467581

[b48] Ashcroft F. M. & Gribble F. M. Correlating structure and function in ATP-sensitive K+ channels. Trends Neurosci 21, 288–294 (1998). 9683320 10.1016/s0166-2236(98)01225-9

[b49] Eliasson L., Renstrom E., Ding W. G., Proks P. & Rorsman P. Rapid ATP-dependent priming of secretory granules precedes Ca(2+)-induced exocytosis in mouse pancreatic B-cells. J Physiol 503 (Pt 2), 399–412 (1997). 9306281 10.1111/j.1469-7793.1997.399bh.x PMC1159871

[b50] Fadista J. . Global genomic and transcriptomic analysis of human pancreatic islets reveals novel genes influencing glucose metabolism. P Natl Acad Sci USA 111, 13924–13929 (2014). 10.1073/pnas.1402665111 PMC4183326 25201977

[b51] Hohmeier H. E. . Isolation of INS-1-derived cell lines with robust ATP-sensitive K+channel-dependent and -independent glucose-stimulated insulin secretion. Diabetes 49, 424–430 (2000). 10868964 10.2337/diabetes.49.3.424

[b52] Agarwal V., Bell G. W., Nam J. W. & Bartel D. P. Predicting effective microRNA target sites in mammalian mRNAs. Elife 4, doi: 10.7554/eLife.05005 (2015). PMC4532895 26267216

[b53] Huang D. W., Sherman B. T. & Lempicki R. A. Systematic and integrative analysis of large gene lists using DAVID bioinformatics resources. Nat Protoc 4, 44–57, doi: 10.1038/nprot.2008.211 (2009). 19131956

