# Supplementary material for: Elevated miR-130a/miR130b/miR-152 expression reduces intracellular ATP levels in the pancreatic beta cell
Source: Sci Rep. 2017 Mar 23;7:44986. doi: 10.1038/srep44986 (PMC5362944; doi:10.1038/srep44986)
Supplement: Supplementary Information [file srep44986-s1.pdf]

SUPPLEMENTARY INFORMATION

Elevated miR-130a/miR130b/miR-152 expression reduces intracellular ATP levels in the pancreatic beta cell

Jones K. Ofori[1,3], Vishal A. Salunkhe[1,3], Annika Bagge[2,3], Neelanjan Vishnu[2,3], Mototsugu Nagao[1,3], Hindrik Mulder[2,3], Claes B. Wollheim[3,4], Lena Eliasson[1,3*], Jonathan L.S. Esguerra[1,3*]

[1]Islet Cell Exocytosis, Department of Clinical Sciences-Malmö, Lund University, Malmö, 205 02, Sweden
[2]Molecular Metabolism, Department of Clinical Sciences-Malmö, Lund University, Malmö, 20502, Sweden
[3]Lund University Diabetes Centre, Skåne University Hospital, Lund and Malmö, Sweden
[4]Department of Cell Physiology and Metabolism, Faculty of Medicine, University of Geneva, Geneva, 1211, Switzerland
[*]Corresponding authors: jonathan.esguerra@med.lu.se; lena.eliasson@med.lu.se

SUPPLEMENTARY FIGURES

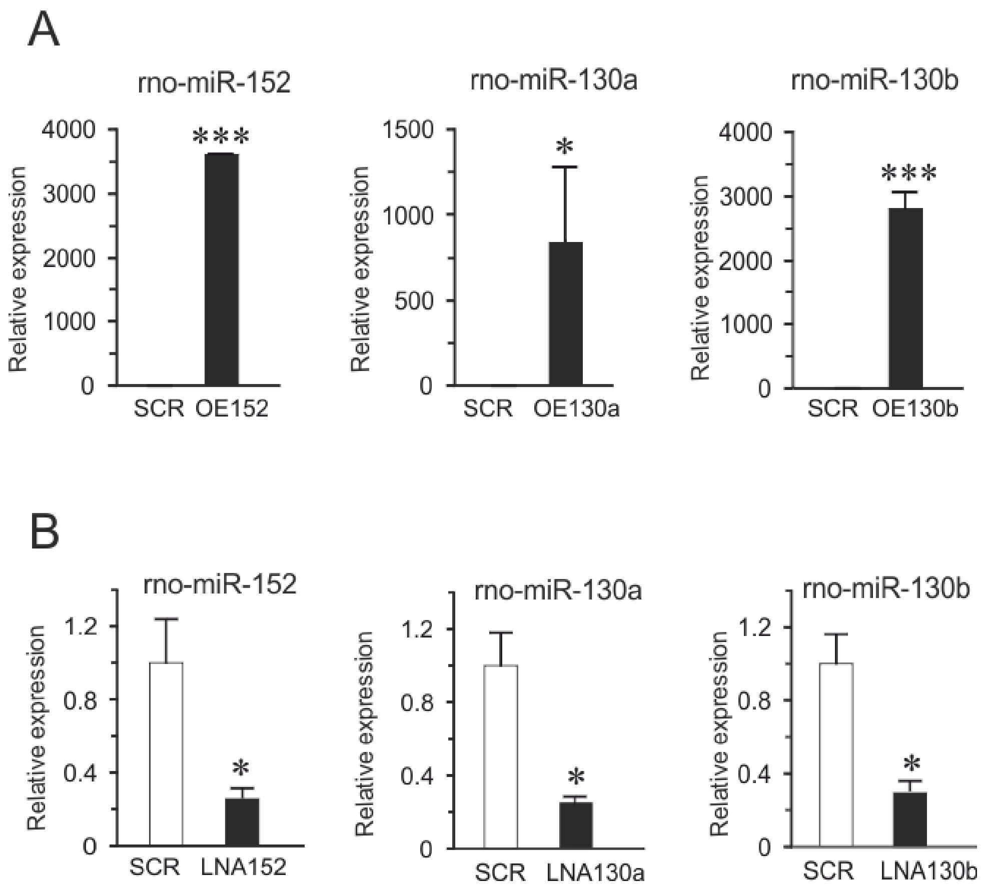

Supplementary figure S1. Expression of miRNAs after over-expression or knock-down of miRNAs in INS-1 832/13. (A) Expression of miRNAs after over- expression in INS-1 832/13 cells. **(B)** Knock-down of miRNAs in INS-1 832/13 Data are presented as mean± SEM of n=3. Students t-test, two tailed; (*) *p*< 0.05, (**) p< 0.01, and (***) p< 0.001.

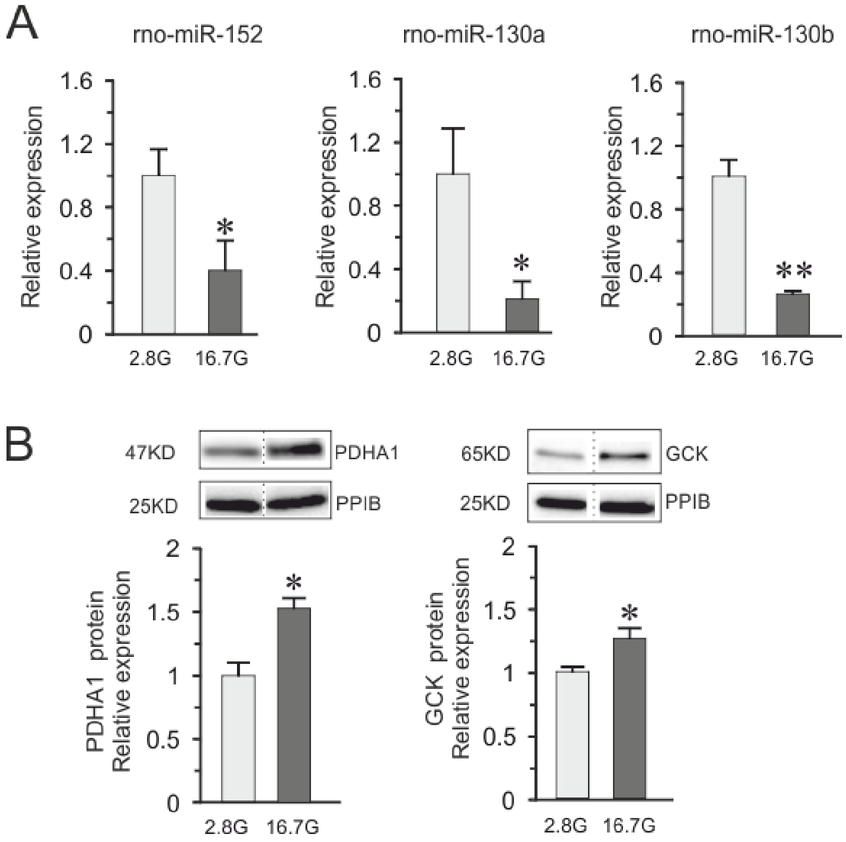

Supplementary figure S2. Glucose-dependence of miRNAs and their putative targets, *Pdha1* and *Gck*. (A) Expression of miRNAs and, **(B)** protein expression levels of PDHA1 and GCK in INS-1 832/13 incubated at 2.8 mM glucose and 16.7mM glucose for 1 hr. In each case the expression at 2.8 mM glucose was used as calibrator. Data are presented as mean± SEM. n=3, (*) $p< 0.05$ and (**) p< 0.01 vs 2.8 mM glucose using Student's 2-tailed test.

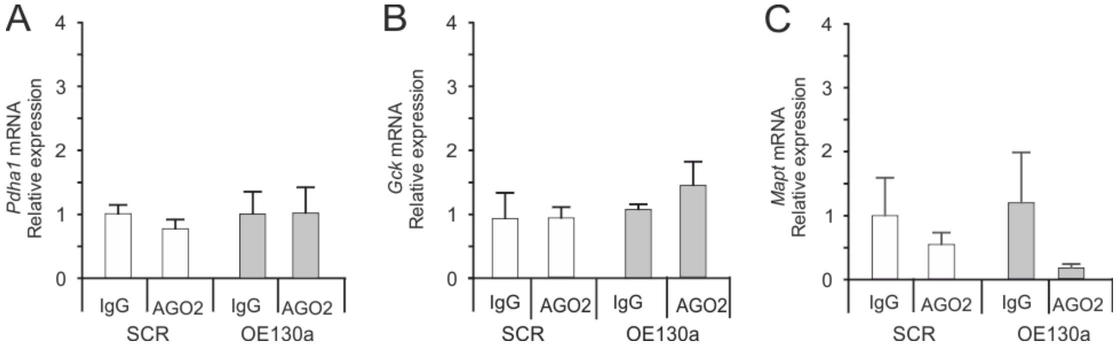

Supplementary figure S3. RIP-AGO2 assay of miR-130a and *Pdha1* or *Gck* targeting. Expression of 3'UTR target region of **(A)** *Pdha1*, **(B)** *Gck*, or **(C)** *Mapt* (non-specific control) after co-immunoprecipitation with anti-AGO2 in OE130a cells. Data are presented as mean ± SEM. n=3, Student's t-test, two tailed.

A Unedited blots for Figure 3A.

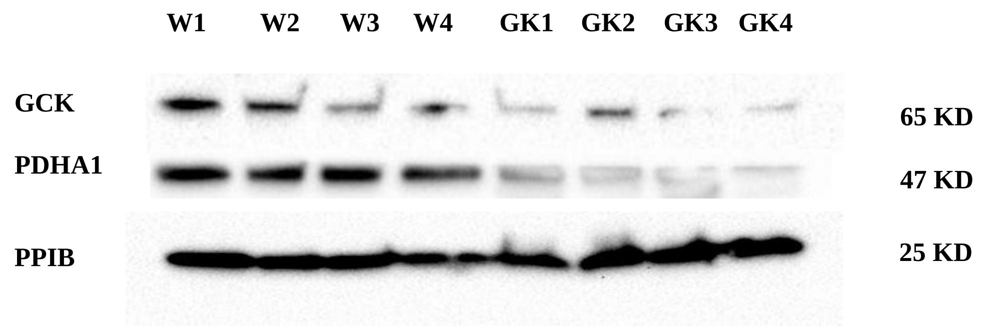

B Unedited blots for Figure 4B. Encircled blots/lanes used as representative in the figure.

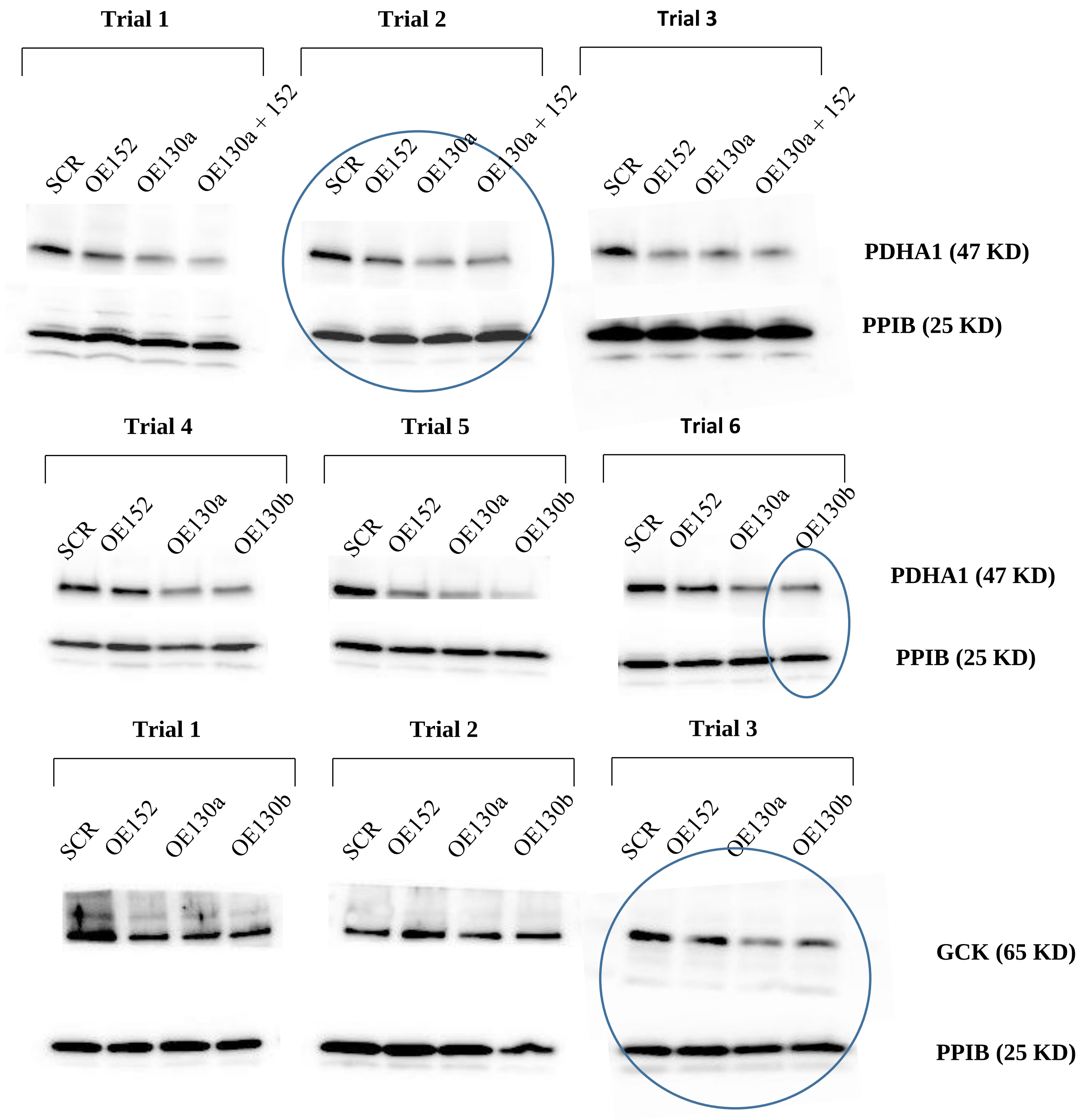

C Unedited blots for Figure 6A. Encircled lanes used as representative in the figure.

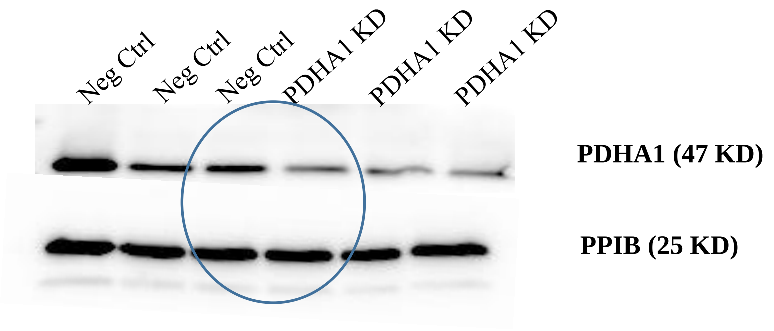

D Unedited blots for Supplementary figure S2B. Encircled lanes used as representative in the figure.

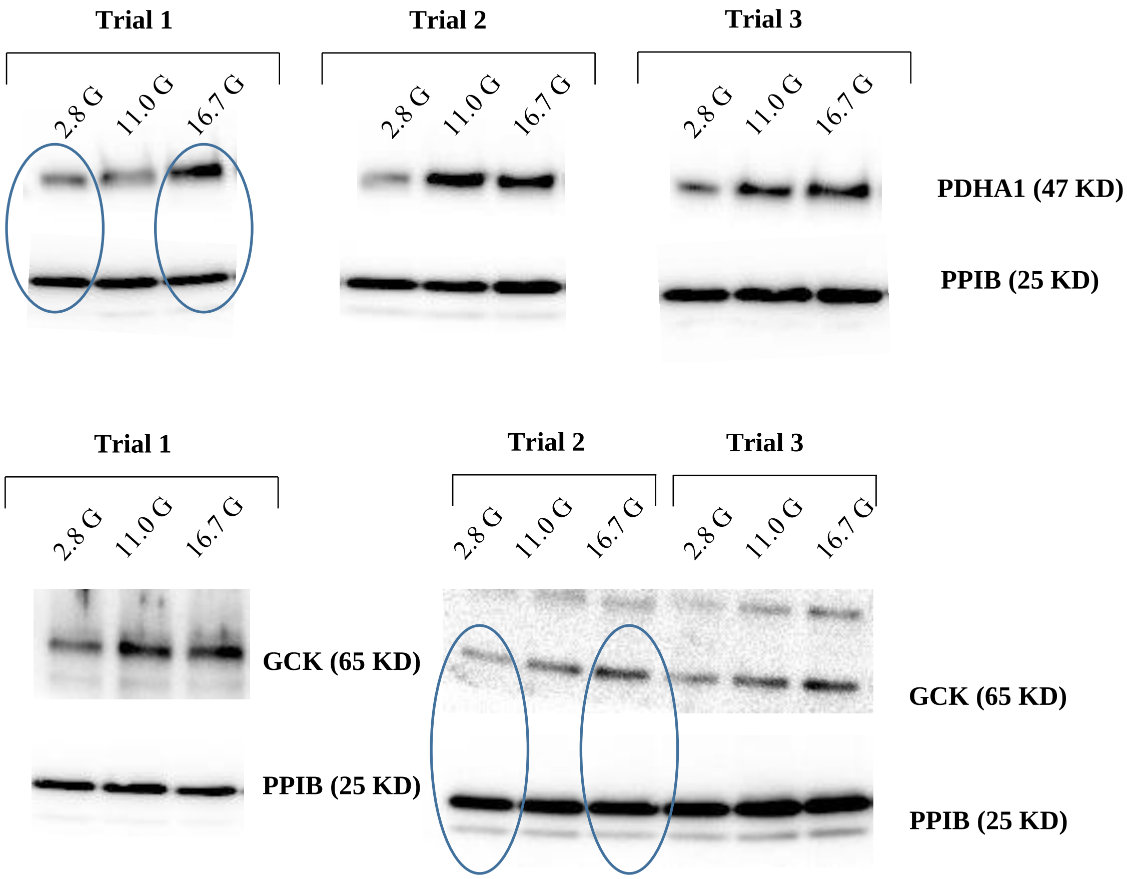

Supplementary figure S4. Unedited blots used in the figures. Blots were cut around the molecular weight of the protein prior to incubation with specific antibodies. Images of whole blots with molecular weight ladders were not acquired prior to cutting.

SUPPLEMENTARY TABLES

Supplementary Table 1. Characteristics of human pancreatic islet donors.

	NGT (n=20)	IGT/T2D (n=22)	p-value
**Gender (Male/Female)**	11/9	8/14	
**HbA1c (%)**	5.4±0.08	6.6±0.14	<0.0001
**Age (years)**	55.8±2.4	57.7±1.8	0.52
**BMI (kg/m2)**	26.3±0.7	27.4±0.8	0.33

NGT: normal glucose tolerant; IGT: impaired glucose tolerant; T2D: type-2 diabetes
Data presented as mean ± S.E.M. Mann-Whitney U test (two-tailed).

Supplementary Table 2. Bioinformatics prediction by miRWalk 2.0 for presence of putative miR-130a/miR-130b/miR-152 binding sites within rat Pdha1/Gck and human PDHA1/GCK.

Web address: http://zmf.umm.uni-heidelberg.de/apps/zmf/mirwalk2/generetsys-self.html

Date of access: November 21, 2016.

(n.d.: no data)

Human	EntrezID	RefseqID	miRNA	MIMATid	miR Walk	Micro t4	miRanda	mir bridge	miR DB	miR Map	miRNA Map	Pictar2	PITA	RNA22	RNA hybrid	Target scan	SUM	Region
GCK	2645	NM_033508	hsa-miR-130a-3p	MIMAT0000425	0	0	0	0	0	0	0	0	0	0	1	0	1	3UTR
GCK	2645	NM_000162	hsa-miR-130a-3p	MIMAT0000425	0	0	0	0	0	0	0	0	0	0	1	0	1	3UTR
GCK	2645	NM_033507	hsa-miR-130a-3p	MIMAT0000425	0	0	0	0	0	0	0	0	0	0	1	0	1	3UTR
GCK	2645	NM_000162	hsa-miR-130b-3p	MIMAT0000691	0	0	0	0	0	0	0	0	0	0	1	0	1	3UTR
GCK	2645	NM_033507	hsa-miR-130b-3p	MIMAT0000691	0	0	0	0	0	0	0	0	0	0	1	0	1	3UTR
GCK	2645	NM_033508	hsa-miR-130b-3p	MIMAT0000691	0	0	0	0	0	0	0	0	0	0	1	0	1	3UTR
GCK	2645	NM_033507	hsa-miR-152-3p	MIMAT0000438	0	0	0	0	0	0	0	0	0	0	1	0	1	3UTR
GCK	2645	NM_000162	hsa-miR-152-3p	MIMAT0000438	0	0	0	0	0	0	0	0	0	0	1	0	1	3UTR
GCK	2645	NM_033508	hsa-miR-152-3p	MIMAT0000438	0	0	0	0	0	0	0	0	0	0	1	0	1	3UTR
PDHA1	5160	NM_001173455	hsa-miR-130a-3p	MIMAT0000425	0	0	0	0	0	0	1	0	0	0	1	0	2	3UTR
PDHA1	5160	NM_001173456	hsa-miR-130a-3p	MIMAT0000425	0	0	0	0	0	0	1	0	0	0	1	0	2	3UTR
PDHA1	5160	NM_000284	hsa-miR-130a-3p	MIMAT0000425	0	0	0	0	0	0	1	0	0	0	1	0	2	3UTR
PDHA1	5160	NM_001173454	hsa-miR-130a-3p	MIMAT0000425	0	0	0	0	0	0	1	0	0	0	1	0	2	3UTR
PDHA1	5160	NM_001173454	hsa-miR-130b-3p	MIMAT0000691	0	0	0	0	0	0	0	0	0	1	1	0	2	3UTR
PDHA1	5160	NM_000284	hsa-miR-130b-3p	MIMAT0000691	0	0	0	0	0	0	0	0	0	0	1	0	1	3UTR
PDHA1	5160	NM_001173455	hsa-miR-130b-3p	MIMAT0000691	0	0	0	0	0	0	0	0	0	0	1	0	1	3UTR
PDHA1	5160	NM_001173456	hsa-miR-130b-3p	MIMAT0000691	0	0	0	0	0	0	0	0	0	0	1	0	1	3UTR
PDHA1	5160	NM_001173454	hsa-miR-152-3p	MIMAT0000438	0	0	0	0	0	0	0	0	0	1	1	0	2	3UTR
PDHA1	5160	NM_001173455	hsa-miR-152-3p	MIMAT0000438	0	0	0	0	0	0	0	0	0	0	1	0	1	3UTR
PDHA1	5160	NM_001173456	hsa-miR-152-3p	MIMAT0000438	0	0	0	0	0	0	0	0	0	0	1	0	1	3UTR
PDHA1	5160	NM_000284	hsa-miR-152-3p	MIMAT0000438	0	0	0	0	0	0	0	0	0	0	1	0	1	3UTR
GCK	2645	NM_000162	hsa-miR-130a-3p	MIMAT0000425	0	0	0	n.d.	n.d.	n.d.	n.d.	n.d.	0	1	1	0	2	CDS
GCK	2645	NM_033507	hsa-miR-130a-3p	MIMAT0000425	0	0	0	n.d.	n.d.	n.d.	n.d.	n.d.	0	0	1	0	1	CDS
GCK	2645	NM_033508	hsa-miR-130a-3p	MIMAT0000425	0	0	0	n.d.	n.d.	n.d.	n.d.	n.d.	0	0	1	0	1	CDS
GCK	2645	NM_033507	hsa-miR-130b-3p	MIMAT0000691	0	0	0	n.d.	n.d.	n.d.	n.d.	n.d.	0	0	1	0	1	CDS

GCK	2645	NM_000162	hsa-miR-130b-3p	MIMAT0000691	0	0	0	n.d.	n.d.	n.d.	n.d.	n.d.	0	0	1	0	1	CDS
GCK	2645	NM_033508	hsa-miR-130b-3p	MIMAT0000691	0	0	0	n.d.	n.d.	n.d.	n.d.	n.d.	0	0	1	0	1	CDS
GCK	2645	NM_000162	hsa-miR-152-3p	MIMAT0000438	0	0	0	n.d.	n.d.	n.d.	n.d.	n.d.	0	0	1	0	1	CDS
GCK	2645	NM_033508	hsa-miR-152-3p	MIMAT0000438	0	0	0	n.d.	n.d.	n.d.	n.d.	n.d.	0	0	1	0	1	CDS
GCK	2645	NM_033507	hsa-miR-152-3p	MIMAT0000438	0	0	0	n.d.	n.d.	n.d.	n.d.	n.d.	0	0	1	0	1	CDS
PDHA1	5160	NM_000284	hsa-miR-130a-3p	MIMAT0000425	0	0	0	n.d.	n.d.	n.d.	n.d.	n.d.	0	0	1	0	1	CDS
PDHA1	5160	NM_001173455	hsa-miR-130a-3p	MIMAT0000425	0	0	0	n.d.	n.d.	n.d.	n.d.	n.d.	0	0	1	0	1	CDS
PDHA1	5160	NM_001173456	hsa-miR-130a-3p	MIMAT0000425	0	0	0	n.d.	n.d.	n.d.	n.d.	n.d.	0	0	1	0	1	CDS
PDHA1	5160	NM_001173454	hsa-miR-130a-3p	MIMAT0000425	0	0	0	n.d.	n.d.	n.d.	n.d.	n.d.	0	0	1	0	1	CDS
PDHA1	5160	NM_001173455	hsa-miR-130b-3p	MIMAT0000691	0	0	0	n.d.	n.d.	n.d.	n.d.	n.d.	0	0	1	0	1	CDS
PDHA1	5160	NM_001173456	hsa-miR-130b-3p	MIMAT0000691	0	0	0	n.d.	n.d.	n.d.	n.d.	n.d.	0	0	1	0	1	CDS
PDHA1	5160	NM_001173454	hsa-miR-130b-3p	MIMAT0000691	0	0	0	n.d.	n.d.	n.d.	n.d.	n.d.	0	0	1	0	1	CDS
PDHA1	5160	NM_000284	hsa-miR-130b-3p	MIMAT0000691	0	0	0	n.d.	n.d.	n.d.	n.d.	n.d.	0	0	1	0	1	CDS
PDHA1	5160	NM_001173456	hsa-miR-152-3p	MIMAT0000438	1	0	0	n.d.	n.d.	n.d.	n.d.	n.d.	0	0	1	1	3	CDS
PDHA1	5160	NM_001173455	hsa-miR-152-3p	MIMAT0000438	1	0	0	n.d.	n.d.	n.d.	n.d.	n.d.	0	0	1	1	3	CDS
PDHA1	5160	NM_001173454	hsa-miR-152-3p	MIMAT0000438	1	0	0	n.d.	n.d.	n.d.	n.d.	n.d.	0	0	1	1	3	CDS
PDHA1	5160	NM_000284	hsa-miR-152-3p	MIMAT0000438	1	0	0	n.d.	n.d.	n.d.	n.d.	n.d.	0	0	1	1	3	CDS

RAT	EntrezID	RefseqID	miRNA	MIMATid	miR Walk	Micro t4	miRanda	mir bridge	miR DB	miR Map	miRNA Map	Pictar2	PITA	RNA22	RNA hybrid	Target scan	SUM	Region
Gck	24385	NM_012565	rno-miR-130a-3p	MIMAT0000836	1	0	1	0	0	1	0	0	0	0	1	1	5	3UTR
Gck	24385	NM_012565	rno-miR-130b-3p	MIMAT0000837	1	0	1	0	0	1	0	0	0	0	1	1	5	3UTR
Pdha1	29554	NM_001004072	rno-miR-130a-3p	MIMAT0000836	0	0	0	0	0	1	0	0	0	0	1	1	3	3UTR
Pdha1	29554	NM_001004072	rno-miR-130b-3p	MIMAT0000837	1	0	0	0	0	1	0	0	0	0	1	1	4	3UTR
Pdha1	29554	NM_001004072	rno-miR-152-3p	MIMAT0000854	1	0	0	0	0	1	0	0	0	0	1	1	4	3UTR
Gck	24385	NM_012565	rno-miR-130a-3p	MIMAT0000836	0	0	0	n.d.	n.d.	n.d.	n.d.	n.d.	0	0	1	0	1	CDS
Gck	24385	NM_012565	rno-miR-130b-3p	MIMAT0000837	0	0	0	n.d.	n.d.	n.d.	n.d.	n.d.	0	0	1	0	1	CDS
Gck	24385	NM_012565	rno-miR-152-3p	MIMAT0000854	0	0	0	n.d.	n.d.	n.d.	n.d.	n.d.	0	0	1	0	1	CDS
Pdha1	29554	NM_001004072	rno-miR-130a-3p	MIMAT0000836	0	0	0	n.d.	n.d.	n.d.	n.d.	n.d.	0	0	1	0	1	CDS
Pdha1	29554	NM_001004072	rno-miR-130b-3p	MIMAT0000837	0	0	0	n.d.	n.d.	n.d.	n.d.	n.d.	0	0	1	0	1	CDS
Pdha1	29554	NM_001004072	rno-miR-152-3p	MIMAT0000854	0	0	0	n.d.	n.d.	n.d.	n.d.	n.d.	0	0	1	0	1	CDS

Supplementary Table 3. Significant Gene ontology (GO)-enriched categories for GOTERM: Metabolic Function of predicted targets unique to (A) miR-152 or (B) miR-130a/b.

GO enrichment performed using The Database for Annotation, Visualization and Integrated Discovery (DAVID) v6.7 web server.

Web address: https://david-d.ncifcrf.gov/

Date of access: 22 November 2016.

(A). Significant GO-enriched categories of targets with unique binding sites for miR-152.

Category	Term	Count	%	PValue	Genes	List Total	Benjamini corrected Pvalue
GOTERM_MF_DIRECT	GO:0005515~protein binding	197	10.3	1.33E-09	NOG, SYT4, PDLIM5, VPS52, VPS53, ADORA1, PGR, HOOK1, BAK1, PICALM, TRAK2, WNK4, CHRNA7, SYK, F11R, SCN2B, ABCB11, SYNJ2BP, PDYN, PPARGC1A, TOX3, HES1, PIAS3, SIPA1L1, MAPK3, RYR2, PRDM2, PIAS2, EIF2AK2, BIN1, CDK5R1, MOB4, ALDOC, CACNB2, RIMS2, STX18, DMD, GIT1, GNAT1, DVL3, KIF3A, SMAD9, NDFIP1, FDXR, SMAD3, YTHDC1, KCNK3, PTPN11, KCNJ8, MAP2, CHRNB2, BEGAIN, NCOR1, SLC9A1, KCNJ16, RTN4, PPARA, LZTS3, TOLLIP, TRPV2, KCNIP3, NR2F2, ERRFI1, FGF2, SCN10A, CEP112, STX1A, STX3, RPH3AL, ARHGEF15, ARHGEF9, SLIT1, SLIT2, MYRIP, IL18BP, GNAQ, SH3KBP1, SIAH2, SNAP29, MYO5A, ATG12, USP2, FHL2, KLC2, ZBTB16, SORBS1, KLC1, KIRREL, ABCD3, APBA2, EXOC3, VMP1, KCNE3, DTNA, UBXN1, DLGAP2, YWHAB, ATP1A3, ATP1A2, CAPN2, ELAVL4, MEF2D, FYN, TENM2, HTR2A, GNA13, CAST, TSPO, SLC6A1, CASK, TBP, MAF1, ANK2, TIAM1, SLC2A3, SLC2A1, TUBG1, DDX20, KCND3, ACTN4, STMN2, RELA, BAIAP2, PRKAB1, PPP1CB, NCAM1, EP300, TIMELESS, RASGRF2, AKAP5, ERC2, CAV1, PANX1, MRAS, KCNA1, CLU, SNX1, SRF, EIF3A, MUC2, MYO1A, EPAS1, KCNB1, PODXL, OMP, FSCN1, ITGA3, PPIF, HDAC4, CDKN1A, PRICKLE1, P2RX1, BBC3, FAAH, DNMT1, IKBKB, PEX3, PAWR, FOS, CASP9, PAK1, LBR, SLC1A1, AKT2, DLG1, SOX11, ARHGAP27, LEF1, UBE2I, ESR2, TIMM23, STOM, KIF1A, KIF1B, CRKL, CARM1, KPNA1, PPP2R2A, DCC, PRKCZ, CABP1, APLP2, HSPA2, TAP1, PPP1R12A, HSPA4, SH2B1, CAMK2A, VPS39, KAT2A, TAB1, PPP1R9B, ATXN3, CYBB, CABIN1, SLC5A7, SMPD3	1514	1.8E-06

GOTERM_MF_DIRECT	GO:0042803~protein homodimerization activity	115	6.0	9.19E-07	ALS2, NOG, HM13, SYT4, GDF6, LHCGR, PRKG2, TPD52, CBX5, KLHL7, BAK1, ABCB10, CHRNA7, NUDT16, PDXK, STK25, ACTN4, RELA, ZHX2, NTSR1, STK4, TOX3, HES1, EIF2AK1, HNF4A, TIMELESS, FGFR1OP, EXT2, DST, CD226, OLFM4, ACHE, SNX6, STK10, HMGCS1, SNX1, GREM2, SRF, TRIM66, RUNX1, SLC30A8, GIT1, MUC2, NADK2, IKZF2, S100A11, SMAD3, FOXP4, FOXP1, KCNK3, TIGIT, ATF3, FAAH, IKBKG, SYT14, PON1, MZF1, TDG, BEGAIN, IKBKB, TCF12, USH1G, MYO7A, DMRTA2, CCDC103, MAX, CRYL1, DGCR8, PSMC3IP, CAT, NR2F2, DPP4, CHUK, ADAM10, PRTFDC1, COL23A1, PFKM, DMRTB1, SLIT2, RAB11FIP4, STOM, RAB11FIP2, RAB11FIP3, SBF2, NPPC, CARM1, CLN6, TYRP1, SLC39A13, CABP1, DCK, RDX, ZBTB16, DROSHA, SH3GLB1, TAP1, ABCD3, HSD17B4, CAMK2A, ACSL6, FLRT3, PLEK, LRRC41, BIRC5, NPR3, STAT1, ADIPOQ, GJB1, MEF2D, TENM2, TENM3, LRP6, DPYD, ZBTB1, RNF40	1514	6.2E-04
GOTERM_MF_DIRECT	GO:0032403~protein complex binding	60	3.1	1.56E-05	RTN4, PPARA, NOG, GNA11, MYO7A, CASK, MMP3, VPS33A, EPCAM, MAX, ANKRD54, BAG3, ATP6V0D1, CIITA, TUB, KIF11, ACTN4, RELA, RPTOR, HES1, NCOA2, EP300, NPTXR, GNAQ, SIPA1L1, PI4K2A, PEX26, AKAP5, RAD18, CPD, ERC2, BIN1, DYNC1I2, PPP2R2A, MED1, CALY, ING2, ABI2, MTHFR, DMD, SH2B3, BRK1, HSPA4, SKIL, GIT1, KIF3A, FBN1, HMBOX1, YWHAB, KCTD2, PARK2, TAB1, PPP1R9B, CDKN1A, SYDE1, FYN, TCEB1, RBPJ, RNF40, HTR2A	1514	7.0E-03
GOTERM_MF_DIRECT	GO:0000978~RNA polymerase II core promoter proximal region sequence-specific DNA binding	55	2.9	3.91E-05	PPARA, JDP2, ARNT2, MITF, PAX6, HOXD13, TBP, KCNIP3, PGR, FOS, ZGPAT, MAX, CREB3L2, POU4F2, RBBP4, EGR2, FOXJ2, FOXJ1, RELA, OTX1, LEF1, ESR2, SIX4, MXD1, CARF, EP300, NCOA2, PRDM5, TXK, MED1, DPF2, SRF, MSX2, CHD7, MEIS2, BCL11B, OVOL1, NFAT5, ETV1, SKIL, NFATC2, ETV6, NFATC3, SMAD3, KLF17, STAT1, FOXP4, NKX6-1, GCFC2, FOXP1, MEF2D, ATF3, MZF1, RBPJ, TCF12	1514	1.3E-02

**GOTERM_MF_DIRECT**	**GO:0005524~ATP binding**	**172**	**9.0**	**5.01E-05**	**RNASEL, ADCY5, STK35, ADCY6, CASK, PRKG2, CCT3, ITPKC, DMPK, DDX17, ATP2B4, WNK4, ABCB10, DDX20, ROS1, MAP2K6, SYK, MAP2K5, CIITA, NMNAT2, PDXK, ROCK1, STK25, ABCB11, ABCC12, CDKL2, STK4, MAP4K3, MYO18B, EIF2AK1, PI4K2A, MAPK3, BMP2K, EIF2AK2, KIF26A, GATC, ERBB3, GNE, STK10, PFKFB2, PEAK1, MAP4K2, MAPKAPK2, MYO9A, UHMK1, ACSBG1, QRSL1, CAMKV, TTBK1, P2RY1, LMTK2, NAT10, RUNX1, STK38L, DNAJA3, DNAJA2, NADK2, MYO1A, KIF3A, TAOK2, ATP11B, TRIO, MYO1F, YTHDC2, OXSR1, DDX5, ATP13A4, ATP7A, GLYCTK, IKBKE, DDX55, ATP2A2, P2RX1, EPHA8, KCNJ8, DYRK1B, DYRK1A, GTF2F2, RAD54L2, SMC1A, IKBKB, UBE2E2, UBE2E1, CDC42BPB, CDK17, NUAK1, ATP5B, MYO7A, PRKAG2, DDR2, CAMKK2, ACVR1C, MTHFD1, MCM9, ACVR1B, HSPH1, PDPK1, PAK2, PAK1, CHUK, AKT3, SIK3, CDK13, AKT2, KIF14, ABCE1, KIF11, CAMK1G, LIMK2, PHKG2, UBE2F, CDK6, UBE2I, UBE2H, PFKM, TTF2, IQCA1, TBCK, MCM6, MAST3, TARS, PDIK1L, ACVR2B, KIF1A, KIF1B, PANK3, HIPK1, SNRNP200, HIPK2, TXK, MYO5A, PRKCZ, BRSK2, DCK, MKNK1, CHD7, MAP3K3, HSPA2, STK40, PARS2, TAP1, TOR1B, DHX15, CHD1, ABCD3, ETNK2, HSPA4, CAMK2A, ACSL6, EHD3, MYO5B, ACSL5, TRIP13, CNNM2, DGKQ, FLT1, NLK, MSH5, PDK4, ATP1A3, STRADB, ATP1A2, FZD7, HSP90B1, FYN, HSPA4L, ABCC4, NLRP14, KATNAL1, CIT, IPPK, ATP8A1**	**1514**	**1.3E-02**
GOTERM_MF_DIRECT	GO:0031625~ubiquitin protein ligase binding	49	2.6	5.04E-05	TRAF1, DERL1, HM13, USP2, TOLLIP, CLU, PAX6, ABI2, CALR, TNFRSF1B, CUL5, KCNQ3, BTBD2, RALA, YOD1, PER3, TRAF6, UBXN1, TXNIP, GABARAPL2, EGR2, SMG5, RELA, NLK, TMBIM6, UBE2F, SMAD3, ERLIN1, UBE2I, CASC3, UBE2H, PARK2, PPARGC1A, MFN2, IKBKE, GPI, ATXN3, CDKN1A, GPR37, SIPA1L1, IKBKG, RAD18, FAF2, PIAS2, FAF1, USP25, MPHOSPH8, RNF40, BTBD11	1514	1.1E-02
GOTERM_MF_DIRECT	GO:0030331~estrogen receptor binding	14	0.7	7.96E-05	MMS19, LEF1, DDX5, PPARGC1A, NRIP1, PAGR1, DDX17, NCOA2, PSMC3IP, DNMT1, PIAS2, NCOR1, NSD1, MED1	1514	1.5E-02
GOTERM_MF_DIRECT	GO:0019901~protein kinase binding	65	3.4	1.33E-04	PRC1, ADCY6, PRKAG2, CSPG4, PAX6, CCNE2, PDPK1, KCNQ3, CDKN2B, ANK2, CASP9, TBC1D14, CHRNA7, MSN, PAK1, CRY1, ERRFI1, MAP2K6, SYK, DLG1, CDK13, KIF14, ELP2, KIF11, ADAM10, RELA, PRKAB1, TPX2, PPP1CB, RPTOR, SPDYA, RAB11FIP2, EP300, RCC2, SIPA1L1, FGFR1OP, AKAP5, RYR2, CD226, MYO5A, PRKCZ, CAV1, CDK5R1, SNAP91, PFKFB2, BRSK2, GCSAM, CACNB2, SORBS1, GYS1, PPP1R12A, TRAF6, DNAJA3, TBL2, SMAD3, RICTOR, PARK2, CDC25B, HDAC4, DUSP3, PRLR, TOM1L1, RAD54L2, FAF1, IKBKB	1514	2.2E-02

GOTERM_MF_DIRECT	GO:0004674~protein serine/threonine kinase activity	52	2.7	1.52E-04	CDK17, FAM20A, NUAK1, STK35, ACVR1C, CAMKK2, DMPK, ACVR1B, PDPK1, WNK4, PAK1, BRD4, AKT3, SIK3, CHUK, AKT2, SYK, LIMK2, ROCK1, CDKL2, STK4, MAST3, MAP4K3, PDIK1L, ACVR2B, HIPK1, HIPK2, MAPK3, BMP2K, EIF2AK2, PRKCZ, STK10, MAP4K2, BRSK2, MKNK1, MAPKAPK2, UHMK1, STK40, TTBK1, LMTK2, STK38L, CAMK2A, TAOK2, NLK, TRIO, OXSR1, FZD7, DYRK1B, DYRK1A, CIT, IKBKB, CDC42BPB	1514	2.3E-02
GOTERM_MF_DIRECT	GO:0042826~histone deacetylase binding	24	1.3	1.99E-04	KAT2A, RBBP4, SMG5, RELA, YWHAB, HR, LEF1, PARK2, UHRF1BP1, SRF, NRIP1, CBX5, HES1, TAL1, MEF2D, HDAC4, DNMT1, AKAP8, DDX20, TRAF6, CRY1, NCOR1, KLF4, PHF6	1514	2.7E-02

(B). Significant GO-enriched categories of targets with unique binding sites for miR-130a/b.

Category	Term	Count	%	PValue	Genes	List Total	Benjamini corrected Pvalue
GOTERM_MF_DIRECT	GO:0003700~transcription factor activity, sequence-specific DNA binding	67	6.4	4.28E-06	CREBRF, MEF2C, BACH1, POU6F1, SOX21, THRA, HNF1A, ELF3, GPBP1, TBX20, PPARG, PAX5, TP63, ZEB1, RORA, FOXO3, DMRTA1, ZBTB38, POU4F1, RARB, MKL2, ALX4, ALX1, AR, YY1, ZHX1, FOXN2, SPEN, TRERF1, GRHL2, MYCL, MYT1L, HOXD4, ST18, NR3C2, SOX4, ELK3, MYT1, HOXA3, TSC22D2, REL, HOXA5, NR1D2, POU2F3, POU3F3, RUNX3, ZBTB7B, IKZF4, IKZF3, KLF7, ERG, TCF7, CREBZF, TBX2, CREB1, RFX6, SMAD5, HOXB3, HOXB2, SP1, ZSCAN30, ZIC5, SP4, HIVEP2, NEUROD1, KLF2, NFIA	825	0.004
GOTERM_MF_DIRECT	GO:0004842~ubiquitin-protein transferase activity	34	3.3	4.09E-05	BACH1, XIAP, RNF217, RNF216, TRIM71, ERCC8, ARIH1, ZFP91, TRIM5, G2E3, UBE2D2, KBTBD3, KLHL28, KBTBD8, HECTD2, ANKIB1, RNF11, KLHL24, KLHL42, TRAF7, RNF34, KLHL20, IPP, PELI1, MUL1, BIRC6, TRIM62, TRIM37, MIB1, KCTD10, RNF6, RNF208, KLHL15, RNF19B	825	0.019
GOTERM_MF_DIRECT	GO:0043565~sequence-specific DNA binding	51	4.9	6.42E-05	MEF2C, CREBRF, POU6F1, THRA, HNF1A, PPARG, MAEL, TP63, FOXO3, RORA, ZEB1, DMRTA1, PAX7, MKX, POU4F1, FOXB1, RARB, ALX4, ALX1, AR, YY1, FOXN2, GRHL2, BPTF, HOXC13, HOXD4, NR3C2, ELK3, ARX, HOXA3, REL, NR1D2, HOXA5, POU2F3, GBX2, POU3F3, IKZF4, IKZF3, ERG, TCF7, CREBZF, TBX2, CREB1, HOXB1, HOXB2, SP1, ZSCAN30, ZBTB4, HIVEP2, NEUROD1, KLF2	825	0.020

GOTERM_MF_DIRECT	GO:0005515~protein binding	102	9.8	1.60E-04	TGOLN2, MEF2C, LDHB, SCN3A, GRIK2, GABRB2, LMO7, RORA, CNOT7, SYP, EPC1, DAB2, MYOCD, SND1, CHRNA5, CHRNA4, RAPGEF4, RARB, RAPGEF3, AKIRIN2, EGFR, MYO6, MAGI2, YY1, WNK1, DLL1, GABRR1, RASGRF1, CLIP1, OPHN1, VAMP3, MAPK8, PTGFRN, SH3GL2, UNC13A, KALRN, DRD1, STX7, ERBB4, UMOD, PFN1, GORASP1, HNRNPD, LURAP1, WDR7, EFEMP1, TGFBR2, SNAPIN, ITGA4, SHANK2, NCKAP1, EPHA5, HDAC5, EPHA7, RAP1A, NUTF2, KAT6A, HNF1A, COPS4, PPARG, GJA1, TP63, KCNJ10, ZEB1, CLTC, GJA3, TRPV4, PLCB1, PRKCA, AR, ARHGEF11, GRM5, BTG1, TPPP, RASD1, TG, PARD3, XIAP, FKBP1A, RGS12, PEX19, TAP2, IPCEF1, PRKAA2, TRIP10, SNAP25, RASA1, TES, NCDN, CREB1, SLC12A5, DPYSL2, APPL1, ITPR1, GCK, SP1, CSNK1D, BAX, GFRA1, CALM2, NFIA, DNM2	825	0.037
